# Short-Chain Inulin and Inulin Neoseries Oligosaccharides from Red Onions Enrich Beneficial Gut Taxa and Attenuate Obesity-Related Outcomes in a Rat Model

**DOI:** 10.3390/foods15162841

**Published:** 2026-08-14

**Authors:** Supachawadee Soyprasert, Kritsakorn Saninjuk, Nalapat Leangnim, Kridsada Unban, Chorchat Lalichatsakul, Pattarasritida Phatthapong, Anusorn Lungkaphin, Chartchai Khanongnuch, Apinun Kanpiengjai

**Affiliations:** 1 Program in Biotechnology, Multidisciplinary and Interdisciplinary School, Chiang Mai University, Chiang Mai 50200, Thailand; supachawadee_s@cmu.ac.th; 2 Division of Biochemistry and Biochemical Innovation, Department of Chemistry, Faculty of Science, Chiang Mai University, Chiang Mai 50200, Thailand; nalapat.l@cmu.ac.th; 3 School of Science, Mae Fah Luang University, Chiang Rai 57100, Thailand; kritsakorn.san@mfu.ac.th; 4 Gut Microbiome Research Group, Mae Fah Luang University, Chiang Rai 57100, Thailand; 5 Office of Research Administration, Chiang Mai University, Chiang Mai 50200, Thailand; 6 Division of Food Science and Technology, Faculty of Agro-Industry, Chiang Mai University, Chiang Mai 50100, Thailand; kridsada.u@cmu.ac.th; 7 Department of Physiology, Faculty of Medicine, Chiang Mai University, Chiang Mai 50200, Thailand; chorchat_lali@cmu.ac.th (C.L.); pattarasritida_ph@cmu.ac.th (P.P.); anusorn.lungka@cmu.ac.th (A.L.); 8 Functional Foods for Health and Disease, Department of Physiology, Faculty of Medicine, Chiang Mai University, Chiang Mai 50200, Thailand; 9 Center of Excellence in Agricultural Innovation for Graduate Entrepreneur, Maejo University, Chiang Mai 50290, Thailand; chartchai_knn@mju.ac.th

**Keywords:** red onions, short-chain fructooligosaccharides, prebiotics, gut microbiota, obesity

## Abstract

Prebiotic-mediated gut microbiota modulation is a promising approach for obesity management. Red onions have been identified as a potential source of inulin and inulin neoseries oligosaccharides. This study evaluated the in vivo effects of short-chain inulin and inulin neoseries oligosaccharides (SCIINOs) on the fecal microbiota of obese rats and assessed their potential role in obesity attenuation. Male Wistar rats were fed a high-fat diet (HFD) for 16 weeks to induce obesity, while control rats received a normal diet (ND). From weeks 16 to 24, HFD-fed obese rats were randomly assigned to four groups: SCIINOs at 0.5 g/day, SCIINOs at 1 g/day, atorvastatin (10 mg/kg/day), or no intervention. Obesity status was confirmed by increased body weight, total cholesterol (TC), low-density lipoprotein cholesterol (LDL-C), and higher food and energy intakes. SCIINOs were enzymatically hydrolyzed and purified by yeast treatment and chromatographic methods. After 8 weeks of SCIINO administration (1 g/day), the fecal microbiota was markedly enriched in *Bifidobacterium* and *Allobaculum*, whereas *Blautia* abundance significantly decreased. SCIINOs also increased taxa negatively associated with obesity-related parameters, including *Dubosiella*, *Rothia*, *Parabacteroides*, *Eubacterium*, and *Coriobacteriaceae UCG-002*, which correlated with reductions in body weight, triglycerides, TC, LDL-C, and fasting blood glucose, along with increased high-density lipoprotein cholesterol (HDL-C). In parallel, SCIINOs increased fecal acetate and butyrate levels, which was consistent with the enrichment of *Bifidobacterium* and butyrate-producing bacteria. Overall, SCIINOs derived from red onions show promise as a prebiotic candidate for obesity attenuation through selective microbiota modulation and enhanced short-chain fatty acid production.

## 1. Introduction

Obesity is primarily caused by long-term calorie intake that exceeds total energy expenditure, leading to the accumulation of excess energy as adipose tissue [1]. The increasing prevalence of obesity depends on several interrelated factors, particularly high-fat diets and sedentary lifestyles, which are directly linked to caloric intake and physical inactivity. These factors are strongly associated with changes in the diversity and composition of the gut microbiome [2], a complex community of microorganisms, mainly comprising bacteria but also fungi, viruses, and archaea, that are known to inhabit the gastrointestinal tract [3].

Given the growing global health concerns related to obesity, the modulation of the gut microbiome through prebiotic consumption has been proposed as an effective approach for obesity management [4]. Moreover, the intake of prebiotics has been shown to support long-term weight maintenance by promoting beneficial gut bacteria that can regulate metabolic health and energy homeostasis [5]. Among prebiotics, fructooligosaccharides (FOSs) are the most extensively studied in the context of obesity and weight management. Other emerging prebiotics include galactooligosaccharides [6], mannooligosaccharides [7], human milk oligosaccharides [8], resistant starch [9], polyphenols [10], and various other plant-based compounds [11]. In animal models with obesity, inulin-type FOSs, specifically short-chain FOSs, play an essential role in improving glucose homeostasis [12], correcting gut dysbiosis, reducing inflammation [13], decreasing fat accumulation, and inhibiting intestinal absorption of dietary fat [14]. Prolonged FOS intake has been shown to reduce body weight and adiposity, alleviate non-alcoholic fatty liver disease (NAFLD), lower serum cholesterol levels, and improve overall metabolic health [15]. The modulation of gut microbiota through FOSs and other prebiotics is considered an alternative strategy for obesity management. Short-chain fructooligosaccharides (SCFOSs) can selectively stimulate the growth of beneficial bacteria, particularly *Bifidobacterium*, *Lactobacillus*, and *Streptococcus*, thereby promoting the production of short-chain fatty acids (SCFAs), e.g., acetate, propionate, and butyrate, along with other health-promoting compounds. These effects, coupled with a reduction in harmful bacteria, such as *Escherichia-Shigella* and *Bacteroides*, can lead to favorable changes in microbial diversity and composition that support obesity treatment and metabolic regulation [16]. Clinical studies have shown that SCFOSs can modulate gut microbiota composition by selectively increasing the abundance of *Bifidobacterium* and *Anaerostipes*, while significantly decreasing the abundance of *Blautia* and *Ruminococcus*. The results revealed that participants administered SCFOSs exhibited no significant changes in glucose metabolism markers, whereas their body weight remained stable when compared with the placebo. The outcomes of this research have indicated that SCFOSs can encourage gut and metabolic health and provide approaches for preventing or hindering metabolic disorders [17].

Inulin-neoseries oligosaccharides are a subgroup of inulin-oligosaccharides that are characterized by a glucose molecule at the terminal end, which is linked to fructose via a β-(2,6) glycosidic linkage. They are naturally found in various plants and grains [18], with onions identified as a major source [19]. Our previous studies identified red onions as a rich source of both inulin and inulin-neoseries oligosaccharides. To enhance their functional applications as prebiotics, these oligosaccharides were enzymatically converted into short-chain inulin and inulin-neoseries oligosaccharides (SCIINOs). Further purification processes involving fermentation and chromatography approaches have provided SCIINOs with a low content of absorbable sugars, including fructose, glucose, and sucrose [19,20]. In vitro fermentation of purified SCIINOs using fecal inocula obtained from normal, overweight, and obese individuals demonstrated the selective enrichment of *Lactococcus* and a reduction in *Escherichia-Shigella* and *Klebsiella* [21], both of which are associated with gut dysbiosis and pathogenicity [22]. However, in vitro studies can have several limitations, particularly those concerning physiological relevance, as the conditions did not fully mimic complex host–microbiota interactions. Therefore, the functional effects of SCIINOs on obesity are not yet fully understood. This study aimed to evaluate, through an in vivo model, the effects of SCIINOs on the gut microbiota of high-fat diet-induced obese rats and to assess the potential role of SCIINOs in obesity attenuation. The outcomes of this study would provide insight into the development of red onions as a prebiotic product, as well as other red onion-based future food products that could be effectively utilized for obesity prevention and treatment.

## 2. Materials and Methods

### 2.1. Preparation of Short-Chain Inulin and Inulin Neoseries Oligosaccharides

Red onions (*A*. *cepa* L. var. viviparum) were purchased from Muang Mai Market, Chiang Mai, Thailand. Fresh onions were peeled, washed, and chopped into small pieces, and then pressed using a hydraulic press. The resulting red onion juice was used as the source of inulin and inulin neoseries oligosaccharides (IINOs). The juice was heated to inactivate endogenous enzymes, cooled to room temperature, and centrifuged to remove insoluble particles. The resulting clear pinkish liquid was designated as crude red onion juice. The characteristics of the crude red onion juice have been described in our previous study [19]. The crude juice was then quantitatively determined for detectable FOSs, including 1-kestose, neokestose, 1-nystose, and 1-fructofuranosyl nystose, as well as simple sugars (sucrose, fructose, and glucose) by high-performance liquid chromatography (HPLC) [19,21]. Total fructan content was determined with a Fructan HK assay kit (Megazyme, Wicklow, Ireland). Sterile red onion juice was sterilized by autoclaving at 121 °C for 15 min prior to being used. Endo-inulinase, EC 3.2.1.7 (Creative-Enzyme^®^, Shirley, NY, USA) was used to produce short-chain IINOs (SCIINOs), while *Candida orthopsilosis* FLA44.2 was used to selectively remove glucose, fructose, and sucrose from the SCIINO preparation following a previously described procedure [19]. Endo-inulinase (assayed under the following conditions: 0.5% (*w*/*v*) inulin, 37 °C, pH 6.5, and reaction time of 10 min) was prepared at 0.4 U/g total fructans in 100 mL sterile water and passed through a 0.22 µm sterile filter.

A single colony of *Candida orthopsilosis* FLA44.2 was inoculated into a 250 mL Erlenmeyer flask containing 50 mL of yeast extract-malt extract broth (YMB) and incubated at 30 °C on a 120-rpm rotary shaker for 24 h. Then, 1% (*v*/*v*) of this culture was transferred into fresh YMB and incubated under the same conditions for 24 h to prepare the inoculum. A total volume of 0.7 L was harvested aseptically by centrifugation, and the resulting cell pellet was resuspended in 200 mL of sterile crude red onion juice. Approximately 6.7 L of sterile crude red onion juice was transferred to a sterile 10 L stirred tank fermenter (B.E. Marubishi Co., Ltd., Tokyo, Japan). Fermentation was conducted at 37 °C with an aeration rate of 2 vvm and an agitation speed of 150 rpm. No pH control was necessary. An antifoam agent was added as needed. Fermentation was carried out for 96 h, or until the ratio of total SCIINO to total fructans exceeded 0.8 and SCIINO purity exceeded 90%, indicating that the short-chain FOS were the main FOS component and that simple sugars accounted for <10% of the detected carbohydrates.

To harvest SCIINOs, the fermentation broth was heated to 80 °C for 30 min, left to cool at room temperature, and centrifuged at 6000 rpm for 10 min to remove any cells. The supernatant was transferred to a stirred tank containing mixed cation and anion exchange resins (0.3 g/3 mL), and then stirred at 50 rpm for 4 h. The resins were removed by filtration through a cloth sheet. A second resin treatment was performed by adding fresh resins (0.15 g/3 mL) to the filtrate and stirring for 1.5 h. The clarified SCIINO solution was obtained by filtration and centrifugation. It was then concentrated using a rotary vacuum evaporator (50 °C) until the total soluble solids reached ~90%, as measured using a handheld refractometer. Purified SCIINO production was performed across 10 batches. Finally, concentrated SCIINO solution from each batch (purity > 90%) was pooled, mixed to homogeneity, and pasteurized using our laboratory protocol prior to being aliquoted into 50 mL bottles and stored at −20 °C for further use. The resulting SCIINOs contained 473.6 g/L neokestose, 54.2 g/L 1-kestose, 31.0 g/L 1-nystose, and 93.8 g/L 1-fructofuranosylnystose, for a total fructan content of 843.4 g/L. This SCIINOs preparation contained residual fructose and sucrose at concentrations of 36.1 g/L and 36.5 g/L, respectively, while no residual glucose was detected. Accordingly, the purity was calculated to be 94.1%.

### 2.2. Animals and Study Design

Six-week-old male Wistar rats (180–200 g) were obtained from Nomura Siam International, Bangkok, Thailand. Rats were housed two per cage under controlled conditions (25 ± 0.5 °C, 12 h light/12 h dark cycle) with ad libitum access to water and feed. All animal procedures were approved by the Laboratory Animal Care and Use Committee at the Faculty of Medicine, Chiang Mai University (Protocol number 18/2567 (C01-2568); approval date 14 November 2024). After a 1-week acclimatization period on a normal diet (ND), rats were randomly assigned to two groups: ND (n = 10) and high-fat diet (HFD) (n = 40). The diet compositions are shown in Table S1. After 16 weeks, HFD-fed rats were randomly allocated into four subgroups (n = 10 per group): (1) H group (HFD control); (2) HS1 group (HFD + SCIINOs, 0.5 g/day); (3) HS2 group (HFD + SCIINOs, 1 g/day); and (4) HA group (HFD + atorvastatin, 10 mg/kg/day). A simple randomization method is used to allocate subjects into experimental groups. SCIINOs and atorvastatin dosages were selected based on previous studies [13,23] and preliminary optimization experiments conducted in our laboratory, which had demonstrated their efficacy in attenuating obesity-related outcomes in a rat model. The SCIINOs were freshly prepared each day as a solution by dissolving the required dose in distilled water. The solution was administered once daily via oral gavage. The experiment continued until week 24. Food intake was measured daily by weighing the amount of food consumed. Energy intake was calculated by multiplying food intake (g/day) by diet energy density and expressed as kcal/day. At weeks 16 and 24, fecal samples were collected for gut microbiota analysis using high-throughput amplicon sequencing, whereas only samples obtained from week 24 were used for quantification of short-chain fatty acids and related compounds by gas chromatography–mass spectrometry (GC–MS). In addition, tail-vein blood was collected after an overnight fasting period for the measurement of HDL-C, total cholesterol (TC), and triglycerides (TG) contents, as well as fasting blood glucose levels, using enzymatic colorimetric assay kits (Erba Diagnostics, Inc., Miami, FL, USA). LDL-cholesterol (LDL-C) was then calculated using the Friedewald equation [24].

### 2.3. DNA Extraction and Sequencing

Total genomic DNA of each sample was extracted using a QIAamp^®^ PowerFecal Pro DNA kit (Hilden, Germany) according to the manufacturer’s instructions. The genomic DNA was observed for degradation by agarose gel electrophoresis. Absorbance was then measured at 260 (A_260_) and 280 nm (A_280_) using a BioDrop Duo+ Microvolume spectrophotometer (Harvard Bioscience, Inc., Cambridge, UK). The qualified DNA of each sample should have a ratio of A_260_/A_280_ that is within the range of 1.8 to 2.0, together with a minimum amount of 2 µg. All samples were amplified using a set of specific primers connecting barcodes (338F: 5′-ACTCCTACGGGAGGCAGCA-3′ and 806R: 5′-GGACTACHVGGGTWTCTAAT-3′) for enrichment of the V3-V4 region of the 16S rRNA gene. The resulting libraries were pooled and sequenced on the Illumina platform by an established service provider (BMKGene, Beijing, China).

### 2.4. Bioinformatics Analysis

Raw sequencing data were processed using the Quantitative Insights Into Microbial Ecology 2 (QIIME2) pipeline (version 2024.5.0). Adapter and primer sequences were trimmed using the Cutadapt plugin [25]. Quality filtering, denoising, merging, and chimera removal were performed via the DADA2 plugin [26] to generate high-quality Amplicon Sequence Variants (ASVs). ASVs were aligned using MAFFT [27] and used to construct a phylogeny with FastTree [28]. Taxonomic classification was assigned using the classify-sklearn naive Bayes taxonomy classifier [29] against the Silva database (silva-138.2-ssu-nr99, released on 11 July 2024).

Alpha diversity indices, including Observed ASVs, Chao1 richness, Shannon diversity, and Faith’s phylogenetic diversity (PD), were calculated and visualized using the phyloseq package in R. Pairwise comparison of alpha diversity between groups was then assessed using the Kruskal–Wallis test (*p* < 0.05).

Beta diversity was evaluated using multiple dissimilarity matrices, including Bray–Curtis dissimilarity, Unweighted UniFrac, Weighted UniFrac, and GUniFrac. Principal coordinate analysis (PCoA) was performed to visualize community differences using phyloseq. Statistical significance of community composition differences was assessed using Permutational Multivariate Analysis of Variance (PERMANOVA) (*p* < 0.05). Additionally, linear discriminant analysis effect size (LEfSe) [30] was employed to identify differentially abundant taxa between groups, utilizing the Kruskal–Wallis sum-rank test to detect biomarkers. All data visualizations were generated using the ggplot2 version 4.0.3 and heatmap packages version 1.0.12 in the R software environment.

### 2.5. Short-Chain Fatty Acid Extraction from Fecal Samples

Pooled fecal samples were subjected to metabolite analysis, with three pooled samples per group (n = 5). Fecal sample preparation in this study was performed with modifications to a previously described method [31]. Accordingly, approximately 0.3 g of the fecal sample was mixed with 2 mL salt-acid buffer (0.9% NaCl and 120 mmol/L HCl) in a 15 mL tube and vortexed vigorously until homogenized. The homogenates were then centrifuged at 8000 rpm for 10 min, and the supernatant was collected. The supernatant was subsequently extracted with 2 mL of ethyl acetate (ratio 1:1, *v*/*v*). After phase separation, 0.5 mL of the upper phase was transferred to a vial for targeted SCFA analysis using GC–MS. An internal standard, including acetic acid, butyric acid, valeric acid, and propionic acid, was included for SCFA quantification. Targeted SCFAs were analyzed using a GC–MS system equipped with an autosampler (PAL sampler). One microliter of the prepared extract was injected into the GC system. Chromatographic separation was performed using a HP-INNOWax capillary column (30 m × 0.25 mm × 0.25 μm; Agilent Technologies, (Santa Clara, CA, USA) with helium as the carrier gas at a constant flow rate of 1.5 mL/min. The inlet temperature was set at 220 °C, and injections were performed in split mode (1:1). The oven temperature program was set as follows: an initial temperature of 60 °C for 2 min, increased to 150 °C at 5 °C/min, followed by a ramp to 230 °C at 20 °C/min with a final hold for 10 min. The total run time was approximately 34 min. Mass spectrometric detection was performed using electron ionization (EI) at 70 eV, and spectra were acquired in full-scan mode over an *m*/*z* range of 40–250. SCFAs were identified based on retention time matching with authentic standards and comparisons of mass spectra.

### 2.6. Statistical Analysis

GraphPad Prism version 10.6.0 (San Diego, CA, USA) was used for statistical analyses. Accordingly, Welch’s *t*-test was applied to compare mean values between two groups, whereas ordinary one-way analysis of variance (ANOVA) was used to compare mean values among three or more groups. The Benjamini–Hochberg false discovery rate (FDR) method was used to adjust *p*-values for multiple testing at the significance threshold of *q* < 0.05. These parametric tests were used to analyze body weight and other metabolic parameters for comparisons between the N and H groups and among the H, HS1, HS2, and HA groups, respectively. For pairwise comparisons of bacterial relative abundance between two groups, the Mann–Whitney U test was used, whereas the Kruskal–Wallis test was applied for comparisons among three or more groups. Statistical significance was defined as *p* < 0.05.

## 3. Results

### 3.1. Effect of High-Fat Diet on Metabolic Parameters of High-Fat-Diet-Induced Rats

High-fat diet (HFD) feeding successfully induced obesity and metabolic dysfunction in Wistar rats. The H group exhibited significantly elevated body weight compared to the normal diet (ND) control group (*p* < 0.05) (Table 1). Notably, total cholesterol (TC) levels were significantly higher in the H group versus the N group (*p* < 0.05), with corresponding increases in low-density lipoprotein cholesterol (LDL-C). High-density lipoprotein cholesterol (HDL-C) levels remained comparable between groups. An unexpected finding was that triglyceride (TG) levels were paradoxically higher in the N group compared to the H group, which may reflect individual variations during assessment. Food and energy intakes were significantly elevated in the H group compared to the N group (*p* < 0.05). Fasting blood glucose levels showed a modest upward trend in the H group, although differences were not statistically significant. These metabolic parameters, particularly increased body weight, TC, and LDL-C, confirmed successful obesity induction. Consequently, atorvastatin was selected as the positive control due to the marked hyperlipidemia in HFD-fed rats, enabling appropriate assessment of SCIINO efficacy in obesity attenuation.

### 3.2. Effect of High-Fat Diet on Bacterial Fecal Microbiota

The N group exhibited significantly higher bacterial species richness and diversity than the H group, as demonstrated by observed features, Chao1, Shannon, and phylogenetic diversity (PD) indices (*p* < 0.05) (Table 1). This reduction in diversity was further confirmed by Venn diagram analysis, which revealed 867 unique taxa in the N group compared to only 118–151 unique taxa in the H, HS1, HS2, and HA groups (Figure S1). These results indicate that HFD significantly diminished bacterial richness and diversity.

**Figure 1:**
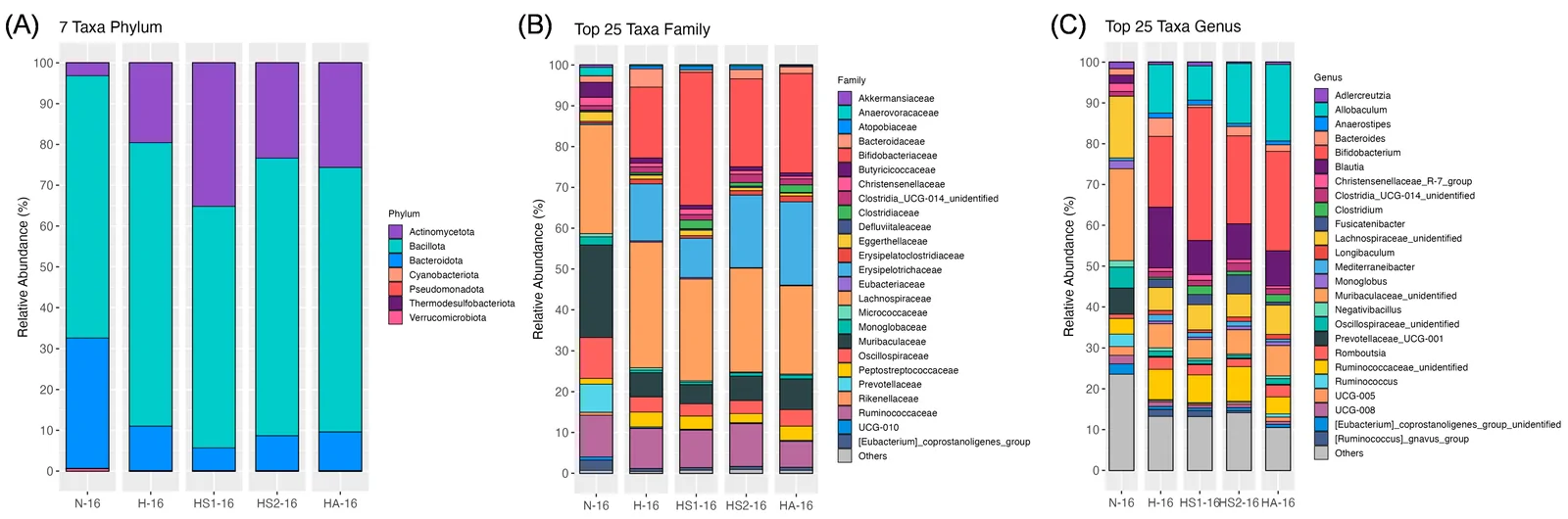
Fecal microbiota composition at week 16. Average relative abundances of bacterial taxa (n = 5) in the fecal samples collected from each group are shown at the (**A**) phylum, (**B**) family, and (**C**) genus levels. Rats were fed either a normal diet (ND; N group) or a high-fat diet (HFD) for 16 weeks. After week 16, HFD-fed rats were allocated to four groups (H, HS1, HS2, and HA) for subsequent intervention experiments.

Taxonomic annotations identified Bacillota (formerly known as Firmicutes), Bacteroidota, and Actinomycetota as three predominant bacterial phyla present in the N and H groups (Figure 1A). The results revealed that the relative abundances of Bacteroidota and Actinomycetota varied depending on dietary type. At the family level (Figure 1B), the N group was dominated by Lachnospiraceae, Muribaculaceae, Ruminococcaceae, Oscillospiraceae, and Prevotellaceae, in descending order of abundance. In contrast, the H group was dominated by Lachnospiraceae, Erysipelotrichaceae, Bifidobacteriaceae, Ruminococcaceae, and Muribaculaceae. Due to insufficient taxonomic resolution, certain genera within the families Lachnospiraceae, Muribaculaceae, Ruminococcaceae, and Oscillospiraceae could not be accurately classified and were therefore annotated as *f_ Lachnospiraceae*, *f_Muribaculaceae*, *f_Ruminococcaceae*, and *f_Oscillospiraceae*, respectively. At the genus level (Figure 1C), *f_Muribaculaceae* and *f_Lachnospiraceae* were the most abundant members of the N group, accounting for 21.0% and 16.1% of relative abundance. In the H group, their relative abundances decreased to 6.4% and 7.2%, respectively. Notably, the top three genera in the H group were *Allobaculum*, *Bifidobacterium*, and *Blautia*. It is suggested that the HFD increased the relative abundance of Actinomycetota, which may have negatively influenced the abundance of Bacteroidota. The Bacillota/Bacteroidota (B/B) ratio was substantially elevated in the H group (8.53) compared to the N group (2.26), indicating a dysbiosis-associated microbiota shift.

**Figure 2:**
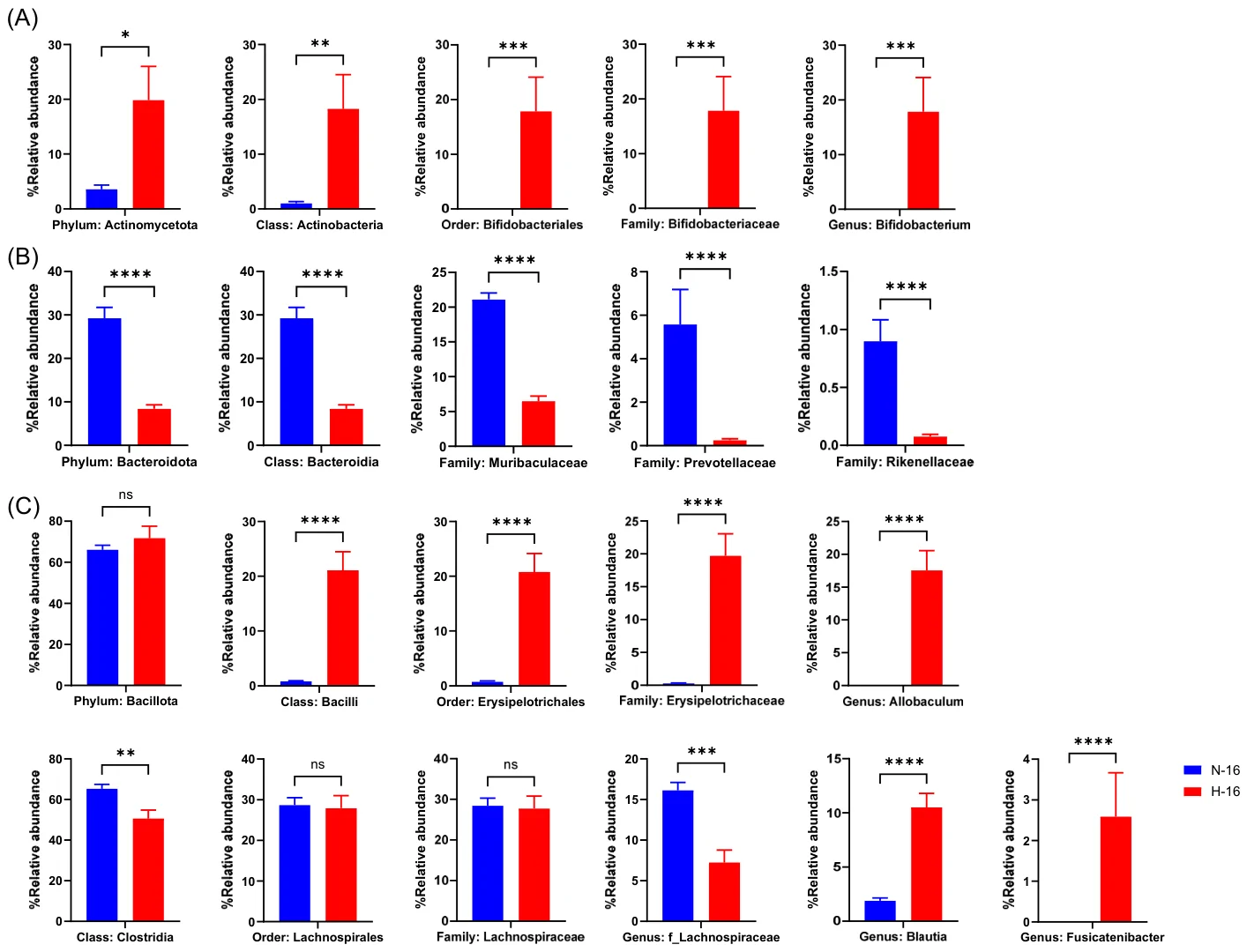
Pairwise comparisons of the three most abundant bacterial phyla, Actinomycetota (**A**), Bacteroidota (**B**), and Bacillota (**C**), and their corresponding lower taxonomic ranks, showing significant differences in relative abundance between the HFD-fed (H) and normal diet (N) groups at week 16. Rats were fed either a high-fat diet (HFD) or a normal diet (ND) for 16 weeks. After week 16, HFD-fed rats were allocated to four groups (H, HS1, HS2, and HA) for subsequent intervention experiments. Data are presented as mean ± SEM (n = 5 for the N group and n = 20 for the H group), where average values of the H group were derived from those of the H, HS1, HS2, and HA groups. Accordingly, *, **, ***, and **** indicate significant differences at *p* < 0.05, *p* < 0.01, *p* < 0.001, and *p* < 0.0001, respectively, while ns indicates no significant difference.

Beta-diversity, analyzed by a PCoA plot based on weighted Unifrac distance, revealed a clear separation between the N and H groups, indicating a significant difference in bacterial community composition (*p* < 0.0009) along the PC1 (67.2%) and PC2 (15.3%) axes (Figure S2). Major alterations in bacterial composition could be explained by changes among the top three phyla (Figure 2). The increased abundance of Actinomycetota in the H group corresponded to higher levels of the class Actinobacteria, order Bifidobacteriales, family Bifidobacteriaceae, and genus *Bifidobacterium* (Figure 2A). Conversely, the higher abundance of Bacteroidota observed in the N group, relative to the H groups, was associated with the class Bacteroidia, order Bacteroidale, and families Muribaculaceae (*f_Muribaculaceae*), Prevotellaceae (*Prevotellaceae UCG-001*), and Rikenellaceae (*Alistipes* and *Rikenella*) (Figure 2B). It was noted that there was no significant difference in the abundance of the genus *Bacteroides*, a member of the family Bacteroidaceae under the phylum Bacteroidota.

Within the phylum Bacillota (Figure 2C), two classes, including Bacilli and Clostridia, exhibited significant differences in comparisons between the N and H groups. The abundance of Bacilli was significantly higher in the H group when compared with the N group (*p* < 0.0001), whereas Clostridia revealed the opposite trend (*p* < 0.01). An increase in Bacilli members was consistent with a significantly higher abundance of the order Erysipelotrichales, family Erysipelotrichaceae, genus *Allobaculum*. Although Clostridia were more abundant in the N group, abundances of the dominant order (Lachnospirales) and family (Lachnospiraceae) under this class were equivalent to those of the H group. It was noteworthy that the top three genera within this family, including *f_Lachnospiraceae*, *Blautia*, and *Fusicatenibacter*, were more prevalent in the H group. These results were consistent with those of the LEfSe analysis (Figure S3), which identified *Anaerostipes*, *Allobaculum*, *Bifidobacterium*, *Blautia*, and *Fusicatenibacter* as the biomarkers of the H group.

**Figure 3:**
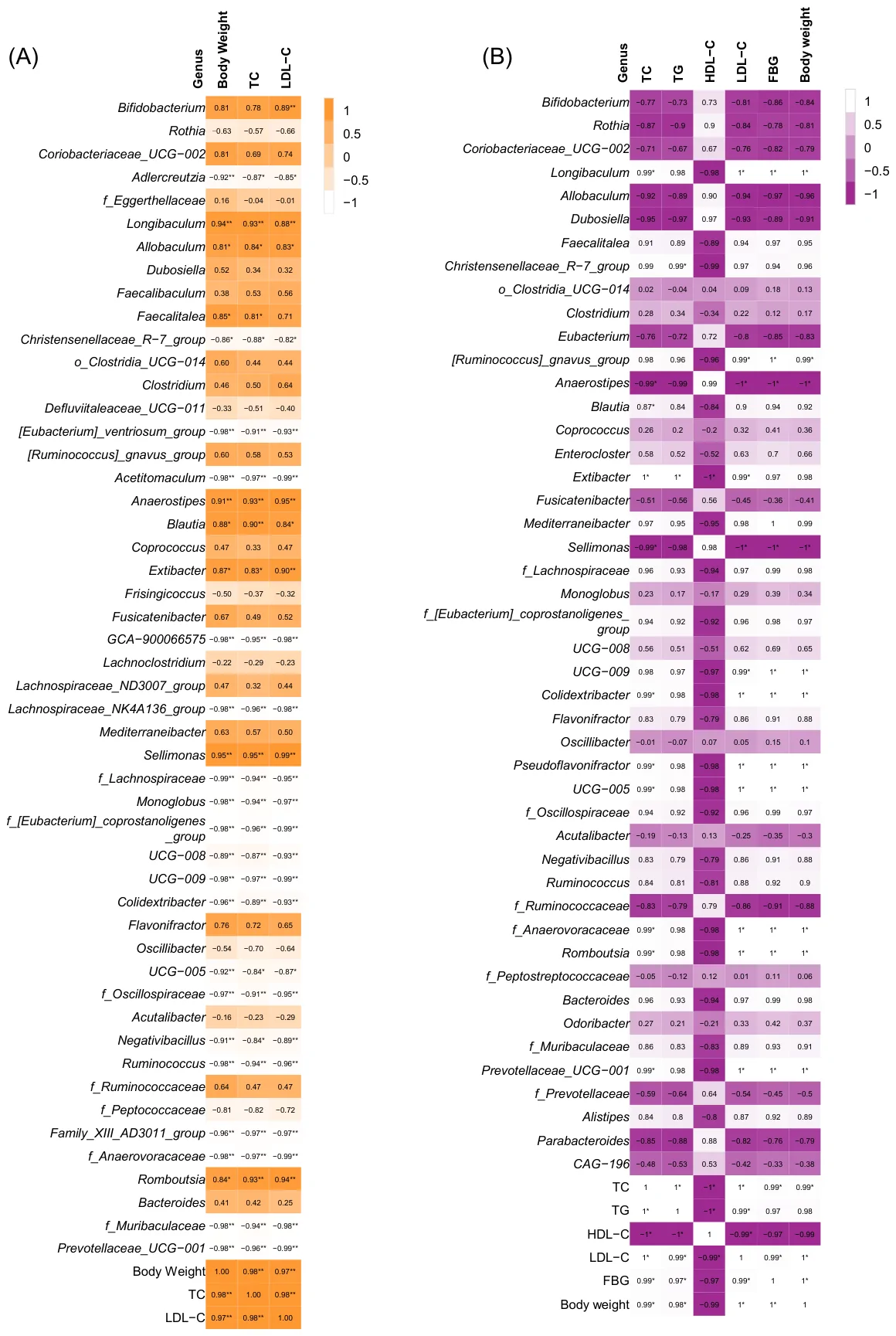
Pearson correlation analysis between key metabolic parameters associated with obesity alleviation and the most abundant genera (relative abundance) in the H, HS1, and HS2 groups at week 16 (**A**) and week 24 (**B**). Accordingly, * and ** indicate statistically significant correlations at *p* < 0.10 and *p* < 0.05, respectively.

To identify bacterial genera that played crucial roles in obesity development, correlation analysis (Figure 3A) was performed between the relative abundance of bacterial genera and metabolic parameters that were significantly affected by a high-fat diet. The results revealed that *Longibaculum*, *Allobaculum*, *Anaerostipes*, *Blautia*, *Extibacter*, *Sellimonas*, and *Romboutsia* were significantly positively correlated with body weight, TC, and LDL-C (*p* < 0.10).

### 3.3. Effect of SCIINOs on Metabolic Parameters of Obese Rats

At week 24, obese rats (H group) exhibited significantly elevated body weight, TG, TC, LDL-C, food intake, energy intake, and fasting blood glucose, along with reduced HDL-C compared to the N group, confirming obesity status (*p* < 0.05). SCIINO administration at both 0.5 g/day (HS1) and 1 g/day (HS2) doses, as well as atorvastatin (HA), significantly reduced body weight, TG, TC, LDL-C, food intake, energy intake, and fasting blood glucose levels compared to untreated obese rats (H group) (*p* < 0.05). A notable finding was substantial improvement in HDL-C levels following intervention. HDL-C increased from 21.80 ± 0.85 mg/dL in the H group to 29.1 ± 1.64 mg/dL (HS1), 27.8 ± 0.75 mg/dL (HS2), and 28.00 ± 1.04 mg/dL (HA) (*p* < 0.05) (Table 2). SCIINO administration dose-dependently improved all obesity-related metabolic parameters, demonstrating efficacy comparable to atorvastatin (10 mg/kg/day), indicating SCIINOs as an effective prebiotic intervention for obesity attenuation.

### 3.4. Effect of SCIINOs on Bacterial Fecal Microbiota

SCIINO administration dose-dependently reduced bacterial richness and diversity (Table 2). The observed features, Chao1, and Shannon indices were significantly lower in the HS2 group (1 g/day SCIINO) compared to the H (untreated obese), HS1 (0.5 g/day SCIINO), N (normal diet), and HA (atorvastatin) groups (*p* < 0.05). However, phylogenetic diversity (PD) indices were similar among H, HS1, HS2, and HA groups, but significantly lower than the N group, indicating treatment-induced shifts toward more closely related species. Venn diagram analysis revealed only 94 taxa shared among all groups. The N group contained the highest number of unique taxa (902), whereas H, HS1, HS2, and HA groups contained 205, 111, 92, and 165 taxa, respectively (Figure S4). This dose-dependent reduction in unique taxa suggests selective bacterial enrichment with higher SCIINO doses.

**Figure 4:**
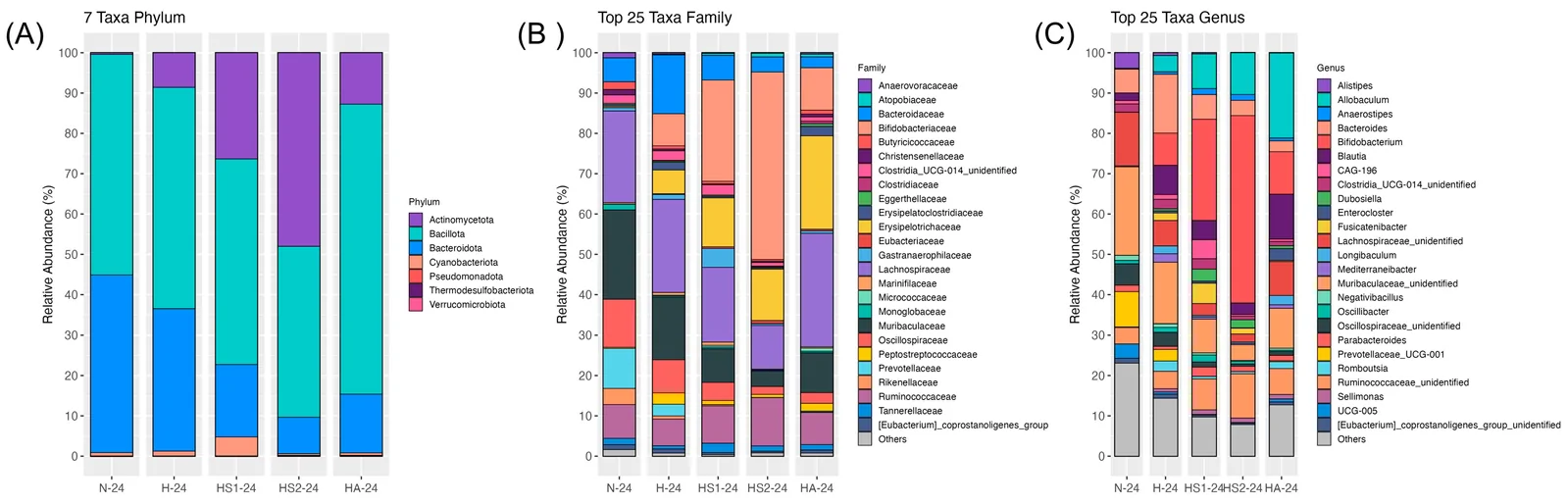
Fecal microbiota composition at week 24. Average relative abundances of bacterial taxa in fecal samples collected from each group are shown at the (**A**) phylum, (**B**) family, and (**C**) genus levels. Rats fed a normal diet served as the negative control (N group), whereas HFD-fed rats served as the positive control (H group). Measurements were obtained after 8 weeks of intervention in obese rats administered SCIINOs at 0.5 g/day (HS1 group) or at 1 g/day (HS2 group), or in those treated with atorvastatin at 30 mg/kg/day (HA group).

Three major phyla dominated all groups: Bacillota, Actinomycetota, and Bacteroidota. SCIINO treatment significantly increased Actinomycetota abundance in HS1 and HS2 groups compared to the H group (Figure 4A, *p* < 0.05), while Bacteroidota significantly decreased across all treatment groups (HS1, HS2, HA; *p* < 0.05). Bacillota remained dominant but without significant differences among groups. Notably elevated B/B ratios were observed: N group (1.26) < H group (1.60) < HS1 (2.90) < HS2 (4.78) ≈ HA (4.84). This dose-dependent increase reflects selective enrichment of Actinomycetota and selective suppression of Bacteroidota.

At the family level, the N group was dominated by Lachnospiraceae, Muribaculaceae, and Oscillospiraceae (Figure 4B). The H group showed Lachnospiraceae, Bacteroidaceae, and Muribaculaceae. Notably, SCIINO-treated groups (HS1, HS2) showed marked shifts with Bifidobacteriaceae as a dominant family, followed by Lachnospiraceae and Erysipelotrichaceae. The HA group displayed Lachnospiraceae and Erysipelotrichaceae as predominant families, with Bifidobacteriaceae at lower abundance. At the genus level, the N group was characterized by high abundance of *f_Muribaculaceae* (22.2%), along with *Prevotellaceae* UCG-001 and *Bacteroides* (Figure 4C). The H group showed Bacteroides (15.7%), *f_Muribaculaceae* (14.8%), *Bifidobacterium* (7.6%), *Blautia* (7.5%), and f*_Lachnospiraceae* (6.5%). *Bifidobacterium* became dominant, with a relative abundance of 25.2% (HS1) and 47.2% (HS2), representing a dose-dependent increase. Other major genera included *f_Ruminococcaceae*, *f_Erysipelotrichaceae*, *f_Muribaculaceae*, *Bacteroides*, and *Blautia*. Bacterial composition resembled the H group but with opposite relative abundance trends, showing elevated *Allobaculum* (Bacilli class) but reduced *Bacteroides*.

Weighted UniFrac distances revealed significant separation among all groups (*p* < 0.001) (Figure S5A). The N group differed significantly from all treatment groups (*p* < 0.05) (Figure S5B). Among treated groups, H significantly differed from HS2 and HA (*p* < 0.05), with a marginal difference from HS1 (*p* < 0.10). HS1 and HS2 showed no significant difference, whereas HS2 and HA differed significantly.

**Figure 5:**
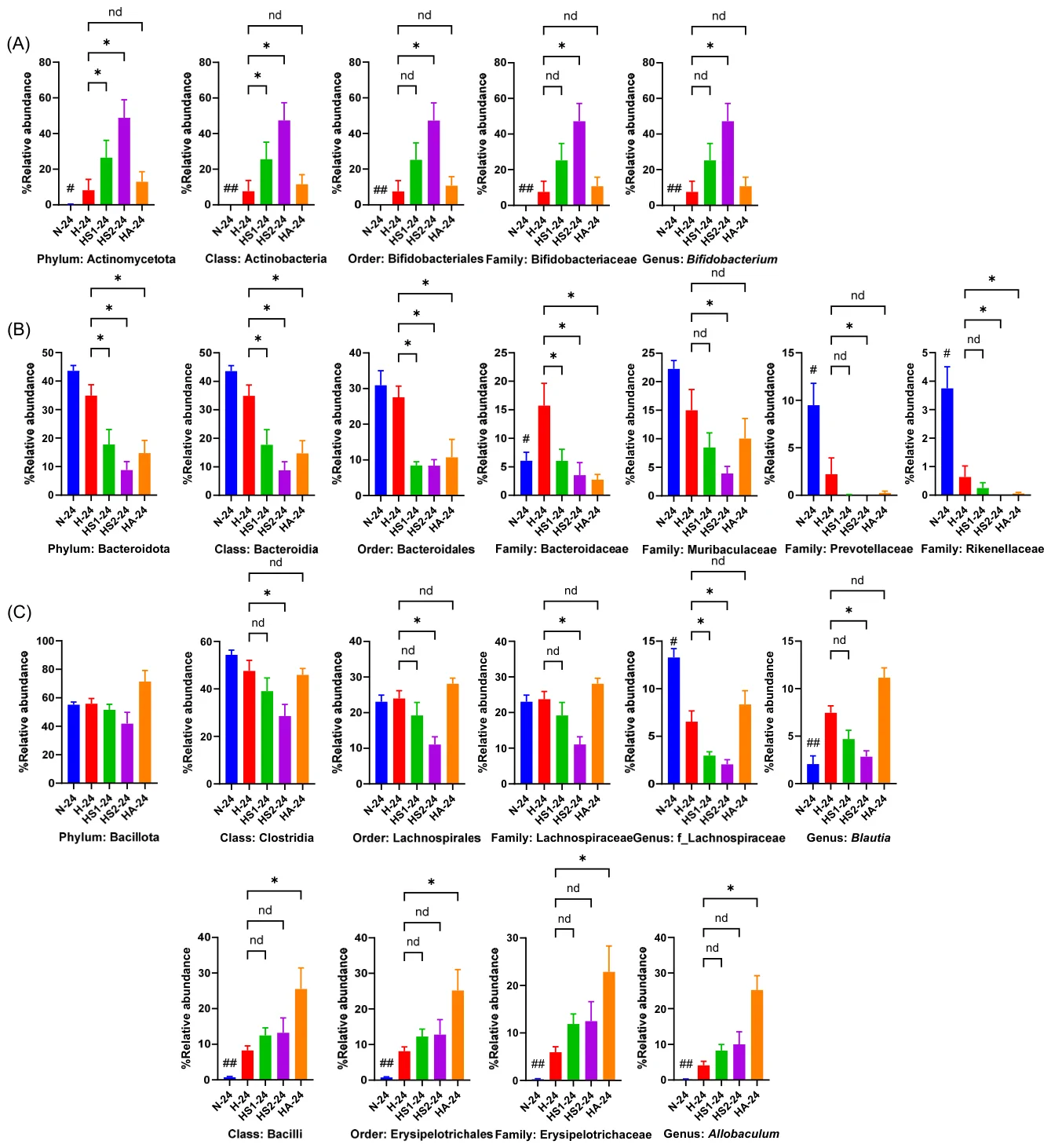
Multiple pairwise comparisons of the three most abundant bacterial phyla, Actinomycetota (**A**), Bacteroidota (**B**), and Bacillota (**C**), and their corresponding lower taxonomic ranks, showing significant differences in relative abundance among all groups at week 24. Rats fed a normal diet served as the negative control (N group), whereas HFD-fed rats served as the positive control (H group). Measurements were obtained after 8 weeks of intervention in obese rats administered SCIINOs at 0.5 g/day (HS1 group), at 1 g/day (HS2 group), or treated with atorvastatin at 30 mg/kg/day (HA group). Pairwise comparisons between the N and H groups were performed using the Mann–Whitney U test, whereas multiple comparisons among the H, HS1, HS2, and HA groups were performed using the Kruskal–Wallis test (with pairwise post hoc comparisons as applicable). Data are presented as mean ± SEM (n = 5). Accordingly, * indicates *p* < 0.05; nd indicates no significant difference; and # and ## indicate significant differences between the N and H groups at *p* < 0.05 and *p* < 0.01.

Microbiota changes were consistent between weeks 16 and 24. The H group showed significantly higher Actinomycetota abundance compared to the N group, while Bacteroidota and Bacillota abundances were similar between groups (Figure 5A). Multiple comparisons among treatment groups (H, HS1, HS2, HA) revealed significant differences for Actinomycetota and Bacteroidota, but not for Bacillota. SCIINO treatment at both 0.5 g/day (HS1) and 1 g/day (HS2) significantly increased *Actinomycetota* abundance compared to the H group, while atorvastatin (HA) showed no significant change (*p* < 0.05). Conversely, Bacteroidota abundance significantly decreased across all treatment groups (HS1, HS2, HA) (Figure 5B). SCIINO administration selectively stimulated Actinomycetota enrichment, consistent with the marked increase in *Bifidobacterium*. Both SCIINOs and atorvastatin suppressed Bacteroidota, particularly the families Bacteroidaceae, Muribaculaceae, Prevotellaceae, and Rikenellaceae. Within Bacillota, opposing trends between the *Clostridia* and *Bacilli* classes resulted in no overall significant phylum-level differences. Clostridia abundance decreased significantly in the HS2 group, associated with reduced Lachnospiraceae (particularly *f_Lachnospiraceae* and *Blautia*) (Figure 5C). Conversely, *Bacilli* abundance only increased in the HA group, driven by elevated *Allobaculum* (Erysipelotrichaceae). SCIINOs selectively increased *Bifidobacterium* and *Allobaculum* while reducing *Blautia*. Atorvastatin partially mimicked SCIINO effects by increasing *Allobaculum* and decreasing *Bacteroides*, but extraordinarily enhanced *Blautia* abundance.

Because alterations in the fecal microbiota were significantly associated with changes in TG, TC, HDL-C, LDL-C, and fasting blood glucose levels, correlation analyses were performed to identify the bacterial genera linked to these metabolic changes. To assess the effect of SCIINO, only the H, HS1, and HS2 groups were analyzed, as atorvastatin was presumed to modulate the fecal microbiota through different mechanisms. TG, TC, LDL-C, and fasting blood glucose levels showed similar correlation patterns, whereas HDL-C showed the opposite pattern (Figure 3B). Increased abundances of *Anaerostipes*, *Sellimonas*, *Dubosiella*, *Allobaculum*, *Rothia*, *Parabacteroides*, *Bifidobacterium*, *Eubacterium*, and *Coriobacteriaceae UCG-002* were significantly correlated with reduced TG, TC, LDL-C, and fasting blood glucose levels, while positively correlated with increased HDL-C (*p* < 0.10). Conversely, *Longibaculum*, *Blautia*, *Extibacter*, and *Romboutsia* remained positively associated with obesity-related metabolic parameters.

### 3.5. Effect of SCIINOs on Short-Chain Fatty Acid Profile of Obese Rats

**Figure 6:**
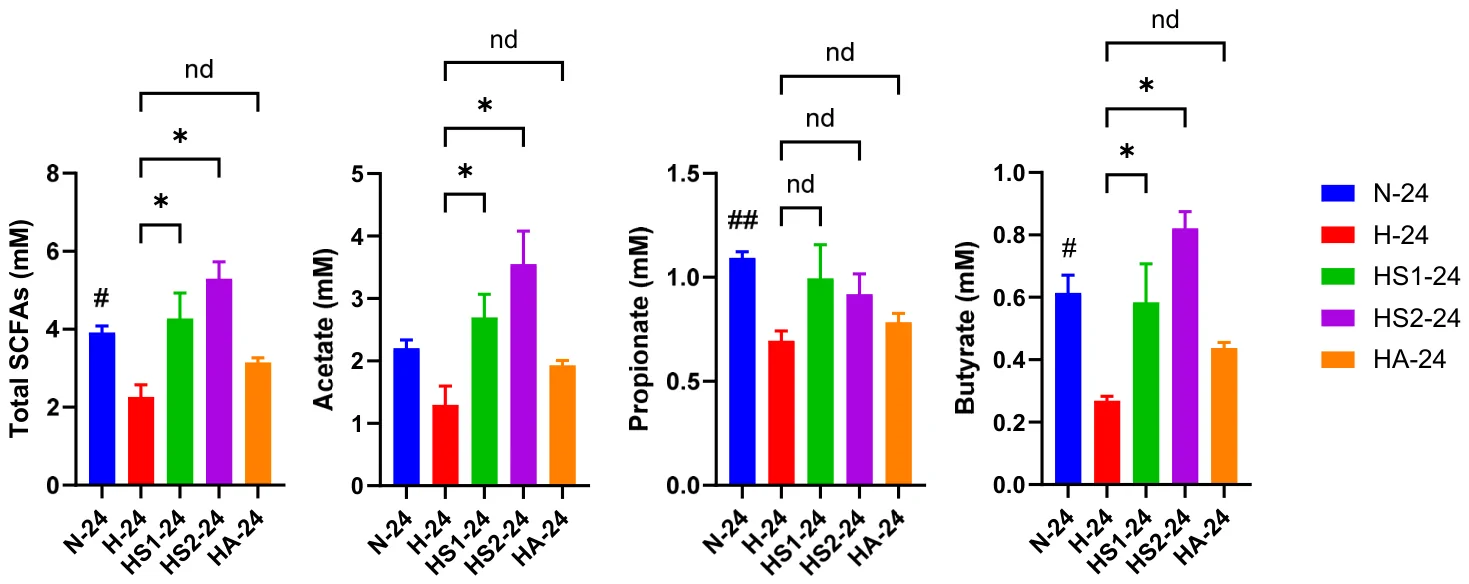
Major short-chain fatty acids (SCFAs) in fecal samples collected at week 24 from normal and HFD-induced obese rats. Rats fed a normal diet served as the negative control (N group), whereas HFD-fed rats served as the positive control (H group). Measurements were obtained after 8 weeks of intervention in obese rats administered SCIINOs at 0.5 g/day (HS1 group), at 1 g/day (HS2 group), or treated with atorvastatin at 30 mg/kg/day (HA group). Data are presented as mean ± SEM. Accordingly, * indicates significant differences among the assessed groups (H, HS1, HS2, and HA); nd indicates no significant differences; while # and ## indicate significant differences between the N and H groups at *p* < 0.05 and *p* < 0.01.

Quantitative analysis results of SCFA content in fecal samples revealed that acetate, propionate, and butyrate were the three major SCFAs (Figure 6). The HS1 (0.5 g/day) and HS2 (1 g/day) groups showed significantly higher total SCFA levels compared to the untreated obese (H) group (*p* < 0.05). Total SCFA content in the H group was comparable to that of the HA group, but the content was significantly lower than that in the N group. The N group had comparable acetate content to the H group, whereas propionate and butyrate contents were significantly higher than those found in the H group. Notably, high acetate and butyrate contents in the HS1 and HS2 groups were significantly higher than those found in the H group. Meanwhile, there were no significant differences in propionate contents among the H, HS1, HS2, and HA groups.

## 4. Discussion

Red onions (*Allium cepa* L. var. viviparum) have emerged as an attractive alternative source of dietary fiber, particularly inulin and inulin neoseries [32]. In addition, red onions naturally contain numerous bioactive compounds, especially phenolic compounds such as anthocyanins and flavonols, which display diverse biological properties including antioxidant, antimicrobial, and anti-inflammatory activities. They have also exhibited anticancer and antidiabetic properties, and ultimately may serve in cardiovascular disease prevention [33]. Our previous work has extensively investigated the utilization of inulin and inulin neoseries from red onions, highlighting their structural characteristics and composition, which differ from those of commercially available FOSs [19]. The short-chain inulin and inulin neoseries oligosaccharides (SCIINOs) prepared from red onions were evaluated as potential prebiotics for obesity prevention and treatment through both in vitro and in vivo experiments.

A validated 16-week high-fat diet (HFD) protocol successfully induced obesity, metabolic syndrome, and a pre-diabetic state in Wistar rats [34]. HFD-fed rats demonstrated significantly elevated body weight, total cholesterol (TC), low-density lipoprotein cholesterol (LDL-C), food intake, and energy intake compared to normal diet (ND) controls. An unexpected finding was elevated plasma triglyceride (TG) levels in the ND group, likely attributable to individual variations during assessment. Collectively, these metabolic parameters confirmed the rats as an appropriate obesity model for evaluating SCIINO efficacy on fecal microbiota composition.

HFD-induced obesity was characterized by reduced bacterial richness and diversity, consistent with previous reports [35,36]. The predominant dysbiotic pattern was increased abundance of *Bacillota* (formerly Firmicutes) coupled with decreased *Bacteroidota*, resulting in an elevated *Bacillota*/*Bacteroidota* (B/B) ratio. However, some studies suggest the strength and relevance of this correlation may be overstated [37,38].

Notably, *Bifidobacterium* (phylum *Actinomycetota*) became the second most abundant genus in obese rats, with stable abundance from week 16 to 24, suggesting it was not selectively stimulated by HFD alone. Similar findings have been reported in other obesity models and in patients with non-alcoholic fatty liver disease [39,40]. HFD induced major microbiota shifts, significantly increasing *Allobaculum*, *Blautia*, and *Fusicatenibacter* abundances while decreasing *f_Lachnospiraceae*, *f_Muribaculaceae*, *f_Prevotellaceae*, and *f_Rikenellaceae*. Correlation analysis identified *Sellimonas*, *Longibaculum*, *Anaerostipes*, *Romboutsia*, *Blautia*, and *Extibacter* as genera positively associated with obesity-related parameters including body weight, TC, and LDL-C levels.

*Blautia* and *Romboutsia* have been identified as important genera positively correlated with obesity indicators, including body weight and serum lipid parameters [41]. However, certain *Blautia* species/strains may contribute to improved lipid metabolism and reduced visceral fat accumulation [42], highlighting the importance of species- and strain-level resolution in microbiota interpretation.

*Allobaculum* abundance was unexpectedly elevated in obese rats, contrasting with some reports suggesting this genus as a target for obesity prevention. While previous studies demonstrate negative correlations between *Allobaculum* and energy intake, body weight, and fasting blood glucose [43], the elevated abundance in this HFD model may represent a baseline component of obesity-associated dysbiosis rather than a protective factor. Similarly, *Extibacter*, while capable of producing secondary bile acids via 7α-dehydroxylation, did not improve metabolic parameters in direct colonization studies [44]. Other obesity-associated genera identified in this study, including *Fusicatenibacter* [45,46], *Longibaculum* [47], *Sellimonas*, and *Anaerostipes* [48], have been linked to obesity phenotypes in previous reports.

After eight weeks of SCIINO administration, the overall metabolic parameters of obese rats were substantially improved and comparable with those observed in the atorvastatin treatment. These improvements were accompanied by characteristic alterations in fecal microbiota composition. Our finding of reduced alpha-diversity with SCIINO treatment aligns with recent clinical and preclinical studies demonstrating that short-chain oligosaccharide supplementation induces selective enrichment of beneficial bacterial taxa at the expense of overall species richness [17]. Specifically, short-chain fructo-oligosaccharides significantly enriched *Bifidobacterium* and *Anaerostipes* while reducing *Blautia*, consistent with our findings.

This apparent contradiction, reduced alpha-diversity coupled with improved metabolic outcomes, reflects functional optimization rather than dysbiosis. The reduced alpha-diversity at the higher SCIINO dose (1 g/day) reflects stronger selective pressure favoring taxa with specialized enzymatic machinery for rapid fermentation of short-chain oligosaccharides [49]. The selective enrichment pattern appears characteristic of short-chain oligosaccharide prebiotics, as demonstrated by previous studies showing that short-chain fructo-oligosaccharides (DP ≈ 7) are more effective than medium- and long-chain variants (DP 14–21) at promoting *Bifidobacterium* dominance.

While the B/B ratio increased with SCIINO treatment, these observations suggest the B/B ratio may not be a robust obesity indicator following dietary or pharmacological intervention because microbiota changes operate at multiple taxonomic levels beyond phylum classification, involve functionally important species-level shifts, are affected by unmeasured phyla, and show inconsistent correlation with obesity attenuation outcomes [50]. Compositional changes at the family, genus, or species level may prove more relevant than the phylum-level B/B ratio for predicting metabolic outcomes.

Several studies demonstrate that gut microbiota regulate lipid metabolism, blood glucose levels, energy metabolism, and inflammatory responses by shaping the bile acid (BA) pool and composition [51,52]. In the intestines, primary bile acids are converted to secondary bile acids through microbial deconjugation, dihydroxylation, oxidation, and epimerization. These secondary BA species modulate host metabolic processes by activating specific nuclear and G protein-coupled receptors in hepatic and intestinal tissues. Additionally, certain secondary and deconjugated bile acids exhibit antimicrobial activity, thereby influencing community structure through selective suppression or promotion of specific taxa while directly regulating host metabolism.

The enrichment of *Bifidobacterium*, *Eubacterium*, and *Parabacteroides* by SCIINO administration may contribute to bile acid deconjugation through production of bile salt hydrolases (BSHs) [53,54,55], key enzymes catalyzing BA deconjugation. Additionally, *Eubacterium* harbors the bai operon, a gene cluster encoding multiple enzymes that mediate 7α-dehydroxylation and secondary bile acid generation [56]. Bile acid oxidation and epimerization involve hydroxysteroid dehydrogenases (HSDHs), which catalyze modifications of primary BA structures to distinctive isomers, activities also reported in certain *Eubacterium* strains [57]. These mechanistic pathways are consistent with the associations observed in the present study.

*Allobaculum* was modestly increased by SCIINO administration but more substantially enriched by atorvastatin treatment. This pattern differs from previous reports in C57BL/6J mice, where high-dose fructo-oligosaccharide administration decreased *Allobaculum* abundance [58]. Atorvastatin decreased *Bacteroidaceae* abundance while increasing *Allobaculum*, differing from prior observations showing atorvastatin increased *Parabacteroides* and *Ruminococcus* [59]. Recent evidence suggests that *Allobaculum* exhibits contradictory roles in obesity depending on dietary intervention, strain specificity, and metabolic context [60,61,62]. It can function both as an obesity-promoting taxon in a dysbiotic state [60], as observed in HFD-induced obesity, and as a protective bacterium when enriched through therapeutic interventions, as demonstrated following SCIINO administration. Diallyl trisulfide (DAT), a bioactive garlic compound, increases *Allobaculum* abundance and reportedly alleviates obesity by inhibiting adipogenesis and lipogenesis. Administration of live or heat-killed *Allobaculum* to HFD-induced obese mice improved glucose tolerance and insulin sensitivity while reducing body weight, TC, and TG [63]. These findings agree with observations that *Allobaculum* enrichment following wasabi intervention correlated with reduced energy intake, body weight, and fasting blood glucose levels [43].

*Blautia* has been established as an obesity-associated genus [64] and was confirmed in this study. Its selective reduction following SCIINO administration (contrasted with the atorvastatin pattern, which partially maintained elevated *Blautia*) suggests SCIINOs selectively enrich beneficial taxa while suppressing those associated with adverse metabolic outcomes. In contrast, atorvastatin acts primarily as a cholesterol-lowering drug with non-specific microbiota effects, indicating distinct mechanistic pathways between the two interventions.

SCFAs are major end products of prebiotic fermentation that have been closely linked to gut microbiota composition and obesity-related metabolic phenotypes. The SCFAs most consistently implicated in obesity attenuation are acetate, propionate, and butyrate, which were also detected in this study. Obese rats exhibited relatively low overall SCFA levels, which were consistent with obesity-associated dysbiosis. We therefore hypothesize that *Bifidobacterium* enriched by SCIINOs acts as a primary acetate producer in the gut, and that acetate may subsequently be converted to butyrate via cross-feeding by butyrate-producing taxa, namely *Eubacterium* [65] and *Faecalibacterium* [66]. These were detected in SCIINO-treated obese rats and showed abundances consistent with SCFA levels (Figure S6). This proposed cross-feeding interaction is supported by recent evidence describing a cooperative metabolism between *Bifidobacterium* and butyrate producers [66].

## 5. Conclusions

This study demonstrates that short-chain inulin and inulin neoseries oligosaccharides (SCIINOs) from red onions represent a promising prebiotic for obesity attenuation. Over eight weeks, SCIINO administration (1 g/day) in HFD-induced obese rats significantly improved metabolic parameters by reducing body weight, total cholesterol, triglycerides, and LDL-cholesterol, while increasing HDL-cholesterol, comparable to atorvastatin treatment. These metabolic improvements were accompanied by selective enrichment of beneficial taxa, including *Bifidobacterium* and *Allobaculum*, while suppressing obesity-associated genera such as *Blautia* and *Romboutsia*. Notably, reduced alpha-diversity, a characteristic feature of short-chain oligosaccharide prebiotics, correlated with improved metabolic outcomes, reflecting functional optimization rather than dysbiosis. The beneficial mechanisms likely involve: (1) *Bifidobacterium*-mediated bile acid deconjugation through bile salt hydrolase production, modulating host lipid metabolism, and (2) increased short-chain fatty acid production, particularly acetate and butyrate, via cross-feeding interactions between *Bifidobacterium* and specialized butyrate producers. SCIINOs demonstrated more targeted microbiota modulation compared to atorvastatin’s non-specific effects. The high-purity SCIINO preparation (94.1%) confirms the feasibility of producing plant-based prebiotics from red onions. However, clinical trials are necessary to validate efficacy in humans and establish SCIINO supplementation as a practical dietary intervention for obesity management.

## Figures and Tables

**Table 1:** Metabolic parameters and alpha-diversity indices at week 16.

Metabolic Parameters	N-16	H-16	HS1-16	HS2-16	HA-16
Body weight (g)	568 ± 17	696 ± 25 **	673 ± 20 *	684 ± 26 **	672 ± 16 **
Triglycerides (mg/dL)	96.37 ± 6.70	75.22 ± 3.42 **	59.27 ± 4.12 ****	72.46 ± 3.45 **	62.61 ± 2.53 **
Total cholesterol (mg/dL)	62.85 ± 2.50	80.81 ± 5.45 **	76.60 ± 1.76 *	76.23 ± 2.45 *	78.88 ± 2.75 **
LDL-Cholesterol (mg/dL)	14.27 ± 2.40	39.77 ± 5.88 **	39.45 ± 5.21 **	35.54 ± 3.33 **	39.36 ± 3.60 **
HDL-Cholesterol (mg/dL)	29.30 ± 1.54	26.00 ± 3.21	25.30 ± 3.65	26.20 ± 3.10	27.00 ± 2.04
Food intake (g/day)	21.07 ± 0.32	23.93 ± 0.71 **	24.07 ± 0.57 **	24.07 ± 0.57 **	23.50 ± 0.70 *
Energy intake (kcal/g)	88.50 ± 1.34	100.5 ± 3.00 **	101.10 ± 2.40 **	101.10 ± 2.40 **	98.70 ± 2.94 *
Fasting blood glucose (mg/dL)	123.43 ± 7.63	144.29 ± 6.02	147.27 ± 3.23	156.76 ± 7.65	146.86 ± 9.21
Alpha-diversity indices					
Observed feature	533.60 ± 35.44	355.40 ± 20.32 *	323.20 ± 23.61 *	314.40 ± 20.89 *	355.80 ± 9.58 *
Chao1	534.60 ± 35.45	366.21 ± 21.91 *	331.26 ± 23.94 *	318.67 ± 20.48 *	361.64 ± 9.13 *
Shannon	5.72 ± 0.08	4.97 ± 0.15 *	4.56 ± 0.25 *	4.75 ± 0.13 *	4.77 ± 0.10 *
PD	176.09 ± 4.43	129.74 ± 3.23 *	126.41 ± 4.39 *	127.68 ± 3.53 *	132.32 ± 3.53 *

Note: Rats were fed either a normal diet (ND; N group) or a high-fat diet (HFD) for 16 weeks. After week 16, HFD-fed rats were allocated to four groups (H, HS1, HS2, and HA) for subsequent intervention experiments. Data are presented as mean ± standard error of mean (SEM) (n = 10 for metabolic parameters and n = 5 for alpha-diversity analysis). Accordingly, *, **, and **** indicate significant differences when compared with the N group at *p* < 0.05, *p* < 0.01, and *p* < 0.0001, respectively.

**Table 2:** Metabolic parameters and alpha-diversity indices at week 24.

Metabolic Parameters	N-24	H-24	HS1-24	HS2-24	HA-24
Body weight (g)	634 ± 22 ^###^	829 ± 46	716 ± 21 *	716 ± 31 *	713 ± 18 *
Triglycerides (mg/dL)	59.64 ± 5.19 ^#^	91.02 ± 9.2	59.56 ± 4.09 **	65.22 ± 3.56 *	67.39 ± 4.14 *
Total cholesterol (mg/dL)	57.35 ± 1.55 ^####^	77.95 ± 0.99	55.23 ± 3.62 ****	57.84 ± 3.72 ***	55.72 ± 1.41 ****
LDL-Cholesterol (mg/dL)	9.54 ± 1.77 ^##^	39.64 ± 2.15	17.70 ± 1.36 ****	18.75 ± 3.12 ****	15.57 ± 2.44 ****
HDL-Cholesterol (mg/dL)	29.70 ± 1.81 ^####^	21.80 ± 0.85	29.10 ± 1.64 ***	27.80 ± 0.75 **	28.00 ± 1.04 **
Food intake (g/day)	21.07 ± 0.32 ^#^	23.14 ± 0.66	19.36 ± 0.33 ****	19.57 ± 0.17 ****	20.14 ± 0.09 ***
Energy intake (kcal/g)	88.50 ± 1.34 ^#^	97.20 ± 2.78	81.30 ± 1.37 ****	82.20 ± 0.73 ****	84.60 ± 0.37 ***
Fasting blood glucose (mg/dL)	148.86 ± 2.75 ^###^	175.00 ± 4.07	151.45 ± 4.61 *	149.82 ± 3.47 *	130.55 ± 9.8 ***
Alpha-diversity indices					
Observed feature	517.00 ± 30.37 ^#^	380.40 ± 33.47	295.20 ± 23.3	242.60 ± 22.49 *	369.60 ± 21.88
Chao1	519.77 ± 30.33 ^#^	385.12 ± 33.15	300.01 ± 23.72	248.99 ± 22.51 *	380.58 ± 22.75
Shannon	5.68 ± 0.07	5.18 ± 0.20	4.62 ± 0.23	4.03 ± 0.18 *	4.92 ± 0.11
PD	172.16 ± 5.34 ^##^	131.33 ± 3.24	121.87 ± 5.91	108.22 ± 8.90	131.2 ± 3.72

**Note:** Rats were fed either a normal diet (ND; N group) or a high-fat diet (HFD) for 16 weeks. After week 16, HFD-fed rats were allocated to four groups (H, HS1, HS2, and HA) for subsequent intervention experiments. Data are presented as mean ± standard error of mean (SEM) (n = 10 for metabolic parameters and n = 5 for alpha-diversity analysis). Accordingly, *, **, ***, and **** indicate significant differences when compared with the N group at *p* < 0.05, *p* < 0.01, *p* < 0.001, and *p* < 0.0001, respectively; while #, ##, ###, and #### indicate significant differences between the N and H groups at *p* < 0.05, *p* < 0.01, *p* < 0.001, and *p* < 0.0001.

## Data Availability

The original contributions presented in the study are included in the article/Supplementary Materials, and further inquiries can be directed to the corresponding author.

## Supplementary Materials

The following supporting information can be downloaded at https://www.mdpi.com/article/10.3390/foods15162841/s1. Table S1: Composition of normal diet (ND) and high-fat diet (HFD); Figure S1: Venn diagram exhibited shard and unique taxa between the N and H groups; Figure S2: Principal coordinates analysis (PCoA) of fecal bacterial communities at week 16 based on weighted UniFrac distances. Fecal samples were collected from rats fed either a normal diet (ND; N group) or a high-fat diet (HFD) for 16 weeks. After week 16, HFD-fed rats were allocated to four groups (H, HS1, HS2, and HA) for subsequent intervention experiments; Figure S3: LEfSe (LDA effect size) analysis between the N and H groups; Figure S4: Venn diagram exhibited shared and unique taxa among the N, H, HS1, HS2, and HA groups; Figure S5: Beta-diversity of fecal bacterial communities at week 24 based on weighted UniFrac distances. (A) Principal coordinates analysis (PCoA) plot and (B) PERMANOVA results. Rats fed a normal diet served as the negative control (N group), whereas HFD-fed rats served as the positive control (H group). Measurements were obtained after 8 weeks of intervention in obese rats administered with SCIINOs at 0.5 g/day (HS1 group), at 1 g/day (HS2 group), or in those treated with atorvastatin at 30 mg/kg/day (HA group). Accordingly, * indicates significant differences at *p* < 0.05; Figure S6: Multiple pairwise comparisons of *Eubacterium* and *Faecalibacterium* abundances across all groups. Pairwise comparisons between the N and H groups were performed using the Mann–Whitney U test, whereas multiple comparisons among the H, HS1, HS2, and HA groups were performed using the Kruskal–Wallis test (with pairwise post hoc comparisons as applicable). Data are presented as mean ± standard error of mean (SEM) (n = 5). * indicates *p* < 0.05; nd indicates no significant difference among H, HS1, HS2, and HA groups; # indicates significant difference between the N and H groups at *p* < 0.05, *p* < 0.01.

## Abbreviations

The following abbreviations are used in this manuscript:

B/B	Bacillota/bacteroidota
BA	Bile acid
BSHs	Bile salt hydrolases
DAT	Diallyl trisulfide
DP	Degree of polymerization
FDR	False discovery rate
FOSs	Fructooligosaccharides
GC-MS	Gas chromatography–mass spectrometry
HDL-C	High-density lipoprotein cholesterol
HFD	High-fat diet
HPLC	High-performance liquid chromatography
HSDHs	Hydroxysteroid dehydrogenases
IINOs	Inulin and inulin neoseries oligosaccharides
LDL-C	Low-density lipoprotein cholesterol
LEfSe	Linear discriminant analysis effect size
NAFLD	Non-alcoholic fatty liver disease
ND	Normal diet
PCoA	Principal coordinate analysis
PERMANOVA	Permutational Multivariate Analysis of Variance
SCFAs	Short-chain fatty acids
SCFOSs	Short-chain frucooligosaccharides
SCIINOs	Short-chain inulin and inulin neoseries oligosaccharides
SEM	Standard error of mean
TC	Total cholesterol
TG	Triglycerides
YMB	Yeast extract-malt extract broth

## References

[1] Safaei M., Sundararajan E.A., Driss M., Boulila W., Shapi’i A. (2021). A systematic literature review on obesity: Understanding the causes & consequences of obesity and reviewing various machine learning approaches used to predict obesity. Comput. Biol. Med..

[2] Aoun A., Darwish F., Hamod N. (2020). The influence of the gut microbiome on obesity in adults and the role of probiotics, prebiotics, and synbiotics for weight loss. Prev. Nutr. Food Sci..

[3] Anto L., Blesso C.N. (2022). Interplay between diet, the gut microbiome, and atherosclerosis: Role of dysbiosis and microbial metabolites on inflammation and disordered lipid metabolism. J. Nutr. Biochem..

[4] Green M., Arora K., Prakash S. (2020). Microbial medicine: Prebiotic and probiotic functional foods to target obesity and metabolic syndrome. Int. J. Mol. Sci..

[5] Ali S., Hamayun M., Siraj M., Khan S.A., Kim H.-Y., Lee B. (2025). Recent advances in prebiotics: Classification, mechanisms, and health applications. Future Foods.

[6] Bajwa A.A., Iqbal S., Sohaib M., Nasir M., Anjum A.A. (2023). Impact of galacto-oligosaccharides on prebiotic potential in the intestinal microbiota fermentation and health status in an animal model. Food Sci. Technol..

[7] Wang H., Zhang X., Wang S., Li H., Lu Z., Shi J., Xu Z. (2018). Mannan-oligosaccharide modulates the obesity and gut microbiota in high-fat diet-fed mice. Food Funct..

[8] Paone P., Latousakis D., Terrasi R., Vertommen D., Jian C., Borlandelli V., Suriano F., Johansson M.E.V., Puel A., Bouzin C. (2024). Human milk oligosaccharide 2′-fucosyllactose protects against high-fat diet-induced obesity by changing intestinal mucus production, composition and degradation linked to changes in gut microbiota and faecal proteome profiles in mice. Gut.

[9] Zeng H., Safratowich B.D., Liu Z., Bukowski M.R. (2025). Resistant starch inhibits high-fat diet-induced oncogenic responses in the colon of C57BL/6 mice. J. Nutr. Biochem..

[10] Guo X., Cheng M., Zhang X., Cao J., Wu Z., Weng P. (2017). Green tea polyphenols reduce obesity in high-fat diet-induced mice by modulating intestinal microbiota composition. Int. J. Food Sci. Technol..

[11] Subramaniyan V., Ravi R.N., Maqsood S., Singh S.K., Chitra V., Dua K., Oliver B.G., Dhanasekaran S., Gupta G., Singh T.G. (2026). The role of plant-derived compounds in anti-obesity drug discovery: A molecular perspective. Curr. Opin. Pharmacol..

[12] Gélineau A., Marcelin G., Ouhachi M., Dussaud S., Voland L., Manuel R., Baba I., Rouault C., Yvan-Charvet L., Clément K. (2024). Fructooligosaccharides benefits on glucose homeostasis upon high-fat diet feeding require type 2 conventional dendritic cells. Nat. Commun..

[13] Pengrattanachot N., Thongnak L., Promsan S., Phengpol N., Sutthasupha P., Tocharus J., Lungkaphin A. (2024). Fructooligosaccharides ameliorate renal injury and dysfunction through the modulation of gut dysbiosis, inhibition of renal inflammation, oxidative stress, fibrosis, and improve organic anion transporter 3 function in an obese rat model. Mol. Nutr. Food Res..

[14] Nakamura Y., Natsume M., Yasuda A., Ishizaka M., Kawahata K., Koga J. (2017). Fructooligosaccharides suppress high-fat diet-induced fat accumulation in C57BL/6J mice. Biofactors.

[15] Bezan P.N., Holland H., Vercesi B.F., Ovídio P.P., Simões L.M.C., Jordão A.A. (2024). Fructooligosaccharides supplementation: A good choice for the prevention and treatment of non-alcoholic fatty liver disease?. Appl. Biosci..

[16] Mo C., Zhou S., Du Z., Huang X. (2025). Impact of fructooligosaccharides on gut microbiota composition and metabolite production: Implications for childhood obesity. PeerJ.

[17] Le Bourgot C., Capronnier O., Graf S., Carton T. (2025). Targeting gut microbiota with short-chain fructo-oligosaccharides prebiotic fibers to support metabolic health in overweight prediabetic adults: A randomized, double-blinded, placebo-controlled study. Front. Nutr..

[18] Leangnim N., Shank L., Chanawanno K., Khanongnuch C., Kanpiengjai A. (2025). Biotechnological production and current feasible applications of neokestose: A review. Carbohydr. Polym. Technol. Appl..

[19] Wongsanittayarak J., Leangnim N., Unban K., Khanongnuch C., Lumyong S., Wongputtisin P., Kanpiengjai A. (2024). Integrated enzymatic hydrolysis of crude red onion extract and yeast treatment for production and purification of short-chain inulin and inulin neoseries oligosaccharides. J. Agric. Food Res..

[20] Aisara J., Wongsanittayarak J., Leangnim N., Utama K., Sangthong P., Sriyotai W., Mahatheeranont S., Phongthai S., Unban K., Lumyong S. (2024). Purification and characterization of crude fructooligosaccharides extracted from red onion (*Allium cepa* var. *viviparum*) by yeast treatment. Microb. Cell Fact..

[21] Wongsanittayarak J., Kanpiengjai A., Leangnim N., Soyprasert S., Unban K., Lumyong S., Khanongnuch C., Wongputtisin P. (2025). In vitro fermentation characteristics of purified short-chain inulin and inulin neoseries oligosaccharides produced from red onions. Foods.

[22] Yang S., Wang Y., Wu Z., Wang D., Zhang X., Hu S., Zhang Q., Bu Y., Liu C., Huang C. (2024). Increased levels of *Escherichia-Shigella* and *Klebsiella* in the gut contribute to the responsivity of placebo analgesia. Neuropharmacology.

[23] Pengrattanachot N., Cherngwelling R., Jaikumkao K., Pongchaidecha A., Thongnak L., Swe M.T., Chatsudthipong V., Lungkaphin A. (2020). Atorvastatin attenuates obese-induced kidney injury and impaired renal organic anion transporter 3 function through inhibition of oxidative stress and inflammation. Biochim. Biophys. Acta Mol. Basis Dis..

[24] Friedewald W.T., Levy R.I., Fredrickson D.S. (1972). Estimation of the concentration of low-density lipoprotein cholesterol in plasma, without use of the preparative ultracentrifuge. Clinial Chem..

[25] Martin M. (2011). Cutadapt removes adapter sequences from high-throughput sequencing reads. EMBnet. J..

[26] Callahan B.J., McMurdie P.J., Rosen M.J., Han A.W., Johnson A.J.A., Holmes S.P. (2016). DADA2: High-resolution sample inference from Illumina amplicon data. Nat. Methods.

[27] Katoh K., Misawa K., Kuma K.i., Miyata T. (2002). MAFFT: A novel method for rapid multiple sequence alignment based on fast Fourier transform. Nucleic Acids Res..

[28] Price M.N., Dehal P.S., Arkin A.P. (2009). FastTree: Computing large minimum evolution trees with profiles instead of a distance matrix. Mol. Biol. Evol..

[29] Bokulich N.A., Kaehler B.D., Rideout J.R., Dillon M., Bolyen E., Knight R., Huttley G.A., Gregory Caporaso J. (2018). Optimizing taxonomic classification of marker-gene amplicon sequences with QIIME 2’s q2-feature-classifier plugin. Microbiome.

[30] Segata N., Izard J., Waldron L., Gevers D., Miropolsky L., Garrett W.S., Huttenhower C. (2011). Metagenomic biomarker discovery and explanation. Genome Biol..

[31] Abbiss H., Shafaei A., Bannister M., Boyce M.C. (2024). Quantitative determination of short chain fatty acids in synthetic feces using gas chromatography with flame ionization detection or mass spectrometry. J. Chem. Educ..

[32] Aisara J., Wongputtisin P., Deejing S., Maneewong C., Unban K., Khanongnuch C., Kosma P., Blaukopf M., Kanpiengjai A. (2021). Potential of inulin-fructooligosaccharides extract produced from red onion (*Allium cepa* var. *viviparum* (Metz) Mansf.) as an alternative prebiotic product. Plants.

[33] Chadorshabi S., Hallaj-Nezhadi S., Ghasempour Z. (2022). Red onion skin active ingredients, extraction and biological properties for functional food applications. Food Chem..

[34] Marques C., Meireles M., Norberto S., Leite J., Freitas J., Pestana D., Faria A., Calhau C. (2016). High-fat diet-induced obesity rat model: A comparison between Wistar and Sprague-Dawley rat. Adipocyte.

[35] Kochumon S., Malik M.Z., Sindhu S., Arefanian H., Jacob T., Bahman F., Nizam R., Hasan A., Thomas R., Al-Rashed F. (2024). Gut dysbiosis shaped by cocoa butter-based sucrose-free HFD leads to steatohepatitis, and insulin resistance in mice. Nutrients.

[36] Tong A., Wu W., Chen Z., Wen J., Jia R., Liu B., Cao H., Zhao C. (2023). Modulation of gut microbiota and lipid metabolism in rats fed high-fat diets by *Ganoderma lucidum* triterpenoids. Curr. Res. Food Sci..

[37] Rodrigues F.G., Ormanji M.S., Meca R., Montenegro H., Cuppari L., de Borst M.H., Heilberg I.P. (2025). Effects of a high-fat diet on gut microbiota and possible implications for bone health in male Wistar rats. Lipids.

[38] Lecomte V., Kaakoush N.O., Maloney C.A., Raipuria M., Huinao K.D., Mitchell H.M., Morris M.J. (2015). Changes in gut microbiota in rats fed a high fat diet correlate with obesity-associated metabolic parameters. PLoS ONE.

[39] Dubinkina V.B., Tyakht A.V., Odintsova V.Y., Yarygin K.S., Kovarsky B.A., Pavlenko A.V., Ischenko D.S., Popenko A.S., Alexeev D.G., Taraskina A.Y. (2017). Links of gut microbiota composition with alcohol dependence syndrome and alcoholic liver disease. Microbiome.

[40] Zhang Z., Liu H., Yu B., Tao H., Li J., Wu Z., Liu G., Yuan C., Guo L., Cui B. (2020). *Lycium barbarum* polysaccharide attenuates myocardial injury in high-fat diet-fed mice through manipulating the gut microbiome and fecal metabolome. Food Res. Int..

[41] Zeng Q., Li D., He Y., Li Y., Yang Z., Zhao X., Liu Y., Wang Y., Sun J., Feng X. (2019). Discrepant gut microbiota markers for the classification of obesity-related metabolic abnormalities. Sci. Rep..

[42] Ozato N., Saito S., Yamaguchi T., Katashima M., Tokuda I., Sawada K., Katsuragi Y., Kakuta M., Imoto S., Ihara K. (2019). *Blautia* genus associated with visceral fat accumulation in adults 20–76 years of age. npj Biofilms Microbiomes.

[43] Thomaz F.S., Tomsett K.I., Panchal S.K., Worrall S., Dekker Nitert M. (2020). Wasabi supplementation alters the composition of the gut microbiota of diet-induced obese rats. J. Funct. Foods.

[44] Streidl T., Karkossa I., Segura Muñoz R.R., Eberl C., Zaufel A., Plagge J., Schmaltz R., Schubert K., Basic M., Schneider K.M. (2021). The gut bacterium *Extibacter muris* produces secondary bile acids and influences liver physiology in gnotobiotic mice. Gut Microbes.

[45] Chen X., Wang Z., Wang D., Kan J. (2023). Effects of resistant starch III on the serum lipid levels and gut microbiota of Kunming mice under high-fat diet. Food Sci. Hum. Wellness.

[46] Wang Y., Tong Q., Shou J.W., Zhao Z.X., Li X.Y., Zhang X.F., Ma S.R., He C.Y., Lin Y., Wen B.Y. (2017). Gut microbiota-mediated personalized treatment of hyperlipidemia using berberine. Theranostics.

[47] Zhao Q., Liu Y., Li M., Zhao L., Wang T., Xiao Y., Wei S., Wu K., Yang J., Wang Y. (2025). Hawthorn pectin mitigates high-fat diet induced hyperlipidemia in mice through promoting *Dubosiella newyorkensis*. Carbohydr. Polym..

[48] Liu Q., Zhao J., Liu S., Fan Y., Mei J., Liu X., Wei T. (2021). Positive intervention of insoluble dietary fiber from defatted rice bran on hyperlipidemia in high fat diet fed rats. J. Food Sci..

[49] Cheng W., Lu J., Li B., Lin W., Zhang Z., Wei X., Sun C., Chi M., Bi W., Yang B. (2017). Effect of functional oligosaccharides and ordinary dietary fiber on intestinal microbiota diversity. Front Microbiol..

[50] Magne F., Gotteland M., Gauthier L., Zazueta A., Pesoa S., Navarrete P., Balamurugan R. (2020). The Firmicutes/Bacteroidetes ratio: A relevant marker of gut dysbiosis in obese patients?. Nutrients.

[51] Jia X., Xu W., Zhang L., Li X., Wang R., Wu S. (2021). Impact of gut microbiota and microbiota-related metabolites on hyperlipidemia. Front. Cell. Infect. Microbiol..

[52] Zhao M., Zhao J., Yang H., Ouyang Z., Lv C., Geng Z., Zhao J. (2025). The bile acid-gut microbiota axis: A central hub for physiological regulation and a novel therapeutic target for metabolic diseases. Biomed. Pharmacother..

[53] Kim G.B., Yi S.H., Lee B.H. (2004). Purification and characterization of three different types of bile salt hydrolases from *Bifidobacterium* strains. J. Dairy Sci..

[54] Liu Y., Pei Y., Wang H., Yang Z. (2025). Lead promoted bile acid deconjugation by modulating gut bacteria encoding bile salt hydrolase (BSH) in *Rana chensinensis* tadpoles. Environ. Pollut..

[55] Qin F., Zhang M., Yang Q., Wu L., Mao T., Zhou X., Li J., Lai M. (2025). Interactions between *Parabacteroides goldsteinii* CCUG 48944 and diet ameliorate colitis in mice via regulating gut bile acid metabolism. iMetaOmics.

[56] Ye H.Q., Mallonee D.H., Wells J.E., Björkhem I., Hylemon P.B. (1999). The bile acid-inducible *baiF* gene from *Eubacterium* sp. strain VPI 12708 encodes a bile acid-coenzyme A hydrolase. J. Lipid Res..

[57] Lee J.Y., Arai H., Nakamura Y., Fukiya S., Wada M., Yokota A. (2013). Contribution of the 7β-hydroxysteroid dehydrogenase from *Ruminococcus gnavus* N53 to ursodeoxycholic acid formation in the human colon. J. Lipid Res..

[58] Mao B., Gu J., Li D., Cui S., Zhao J., Zhang H., Chen W. (2018). Effects of different doses of fructooligosaccharides (FOS) on the composition of mice fecal microbiota, especially the *Bifidobacterium* composition. Nutrients.

[59] Khan T.J., Ahmed Y.M., Zamzami M.A., Mohamed S.A., Khan I., Baothman O.A.S., Mehanna M.G., Yasir M. (2018). Effect of atorvastatin on the gut microbiota of high fat diet-induced hypercholesterolemic rats. Sci. Rep..

[60] Zheng Z., Lyu W., Ren Y., Li X., Zhao S., Yang H., Xiao Y. (2021). *Allobaculum* involves in the modulation of intestinal ANGPTLT4 expression in mice treated by high-fat diet. Front. Nutr..

[61] Deng Y., Liu W., Wang J., Yu J., Yang L.Q. (2020). Intermittent fasting improves lipid metabolism through changes in gut microbiota in diet-induced obese mice. Med. Sci. Monit..

[62] Everard A., Lazarevic V., Gaïa N., Johansson M., Ståhlman M., Backhed F., Delzenne N.M., Schrenzel J., François P., Cani P.D. (2014). Microbiome of prebiotic-treated mice reveals novel targets involved in host response during obesity. ISME J..

[63] Hu Y., Xu Y., Huangfu B., Zhang R., Xu J., Gao R., Wang T., Wang J., Huang K., Luo Y. (2025). *Allobaculum* plays a predominant role in the activation of brown fat tissue by diallyl trisulfide. Food Front..

[64] Chanda W., Jiang H., Liu S.J. (2024). The Ambiguous correlation of *Blautia* with obesity: A systematic review. Microorganisms.

[65] Mukherjee A., Lordan C., Ross R.P., Cotter P.D. (2020). Gut microbes from the phylogenetically diverse genus *Eubacterium* and their various contributions to gut health. Gut Microbes.

[66] Onodera H., Sato Y., Komatsu Y., Yamashita M., Watanabe Y., Kokubo T. (2025). HMOs induce butyrate production of *Faecalibacterium prausnitzii* via cross-feeding by *Bifidobacterium bifidum* with different mechanisms for HMO types. Microorganisms.

