# Supplementary material for: Short-Chain Inulin and Inulin Neoseries Oligosaccharides from Red Onions Enrich Beneficial Gut Taxa and Attenuate Obesity-Related Outcomes in a Rat Model
Source: Foods. 2026 Aug 14;15(16):2841. doi: 10.3390/foods15162841 (PMC13512752; doi:10.3390/foods15162841)
Supplement: Supplementary file 1 [file foods-15-02841-s001.zip › foods-4462090-supplementary.pdf]

Supplementary Material

Short-Chain Inulin and Inulin Neoseries Oligosaccharides from Red Onions Enrich Beneficial Gut Taxa and Attenuate Obesity-Related Outcomes in a Rat Model

Supachawadee Soyprasert [1,2], Kritsakorn Saninjuk [3,4], Nalapat Leangnim [2,5], Kridsada Unban [6], Chorchat Lalichatsakul [7,8], Pattarasritida Phatthapong [7,8], Anusorn Lungkaphin [7,8], Chartchai Khanongnuch [9] and Apinun Kanpiengjai [2,*]

[1] Program in Biotechnology, Multidisciplinary and Interdisciplinary School, Chiang Mai University, Chiang Mai 50200, Thailand
[2] Division of Biochemistry and Biochemical Innovation, Department of Chemistry, Faculty of Science, Chiang Mai University, Chiang Mai 50200, Thailand
[3] School of Science, Mae Fah Luang University, Chiang Rai 57100, Thailand
[4] Gut Microbiome Research Group, Mae Fah Luang University, Chiang Rai 57100, Thailand
[5] Office of Research Administration, Chiang Mai University, Chiang Mai 50200, Thailand
[6] Division of Food Science and Technology, Faculty of Agro-Industry, Chiang Mai University, Chiang Mai 50100, Thailand
[7] Department of Physiology, Faculty of Medicine, Chiang Mai University, Chiang Mai 50200, Thailand
[8] Functional Foods for Health and Disease, Department of Physiology, Faculty of Medicine, Chiang Mai University, Chiang Mai 50200, Thailand
[9] Center of Excellence in Agricultural Innovation for Graduate Entrepreneur, Maejo University, Chiang Mai 50290, Thailand
* Correspondence: Apinun Kanpiengjai, apinun.k@cmu.ac.th

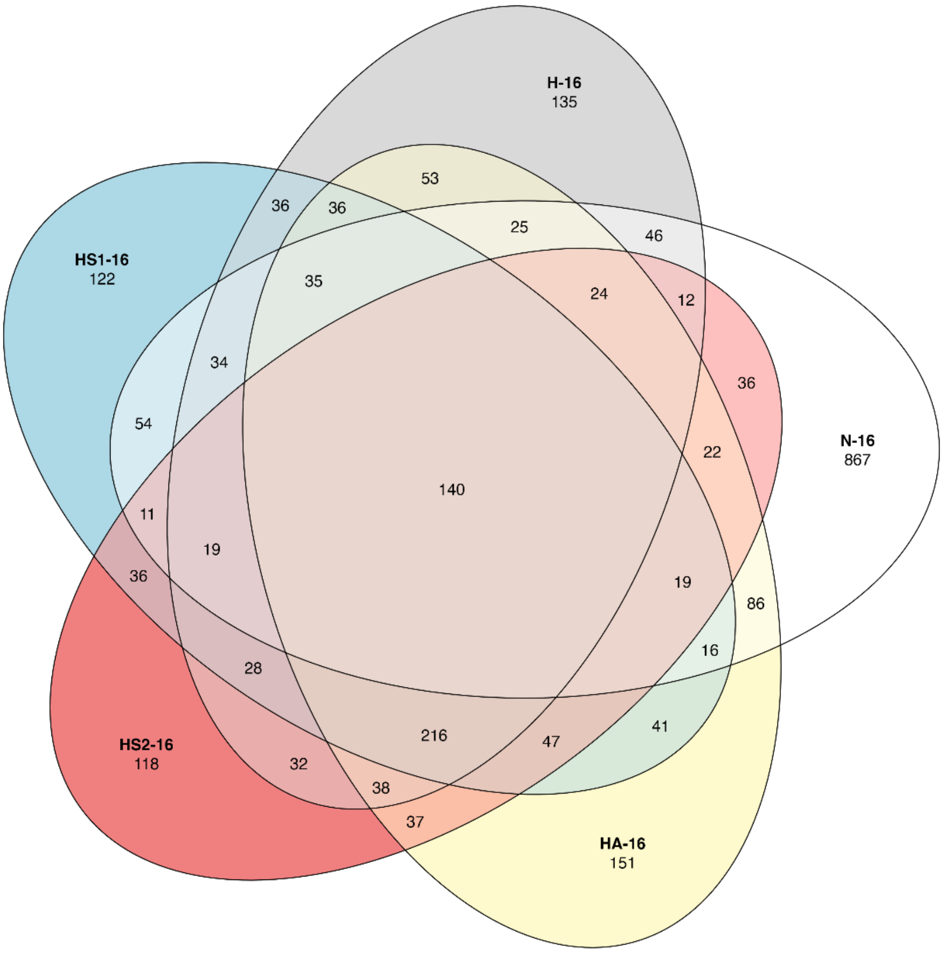

Figure S1. Venn diagram exhibited shard and unique taxa between the N and H groups.

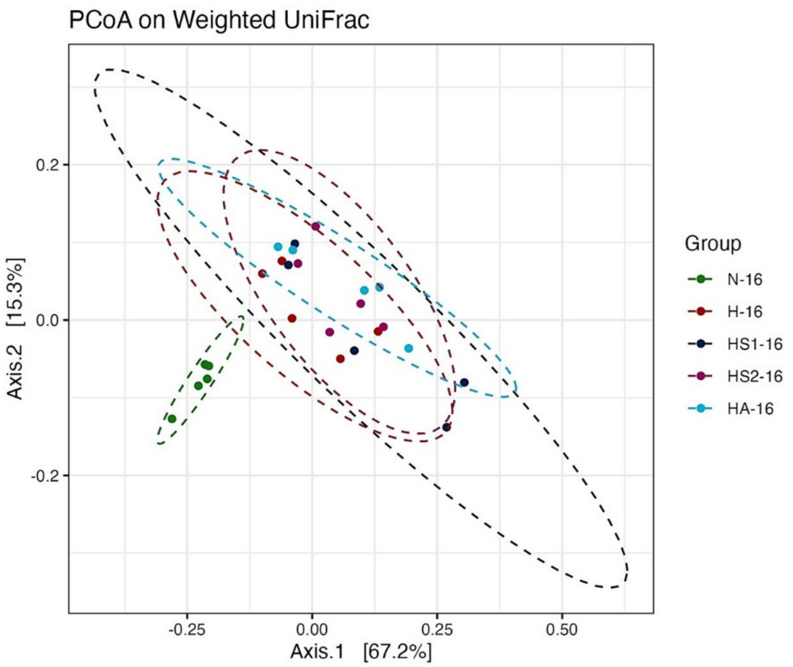

Figure S2. Principal coordinates analysis (PCoA) of fecal bacterial communities at week 16 based on weighted UniFrac distances. Fecal samples were collected from rats fed either a normal diet (ND; N group) or a high-fat diet (HFD) for 16 weeks. After week 16, HFD-fed rats were allocated to four groups (H, HS1, HS2, and HA) for subsequent intervention experiments.

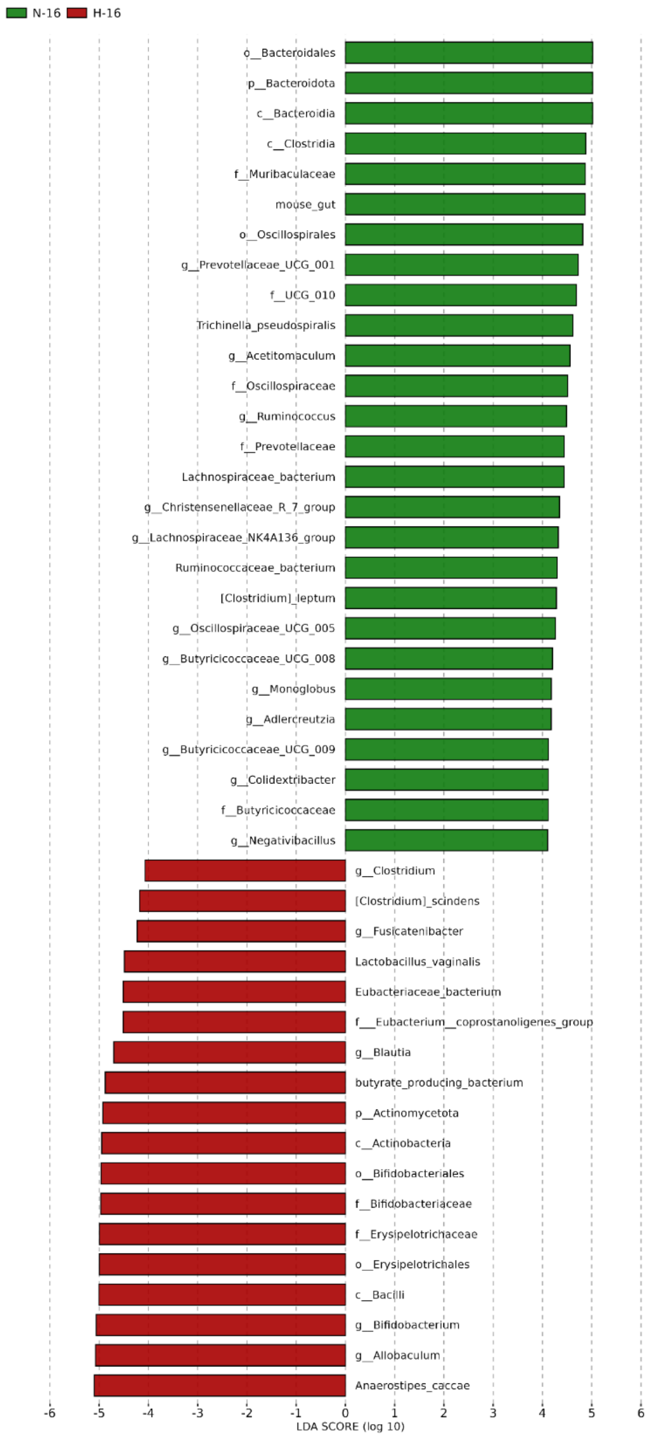

Figure S3. LEfSe (LDA effect size) analysis between the N and H groups.

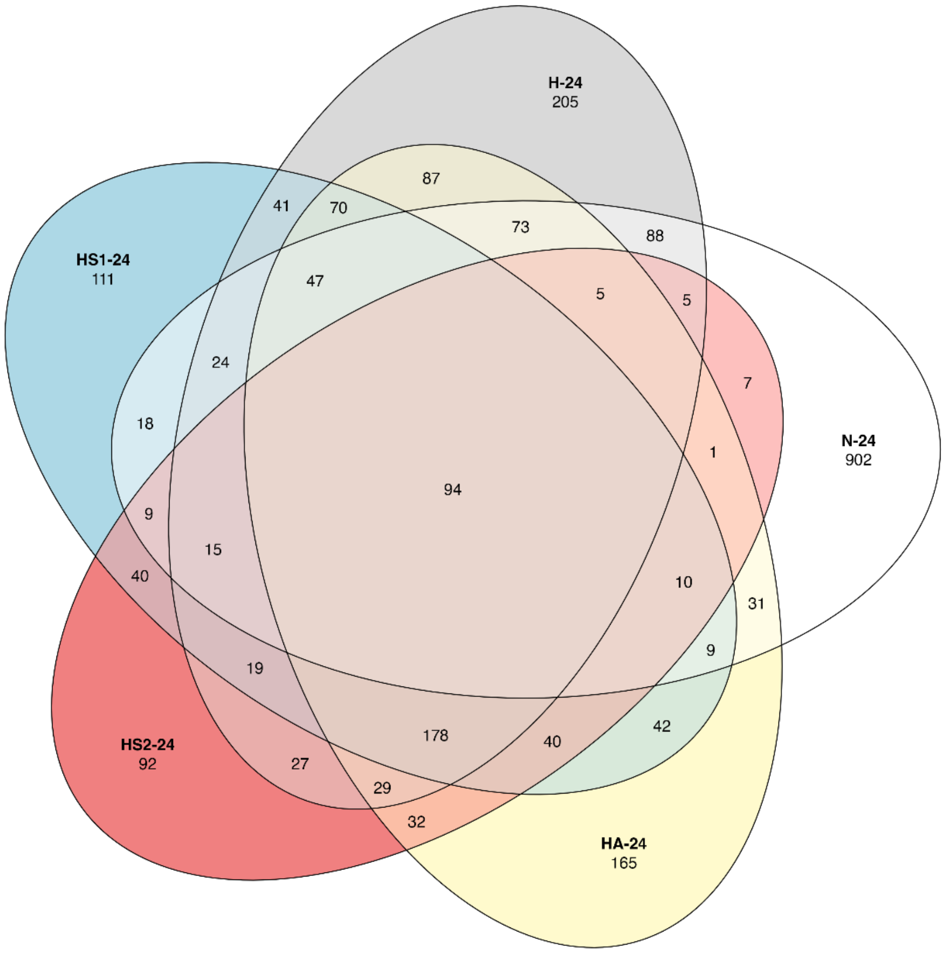

Figure S4. Venn diagram exhibited shared and unique taxa among the N, H, HS1, HS2, and HA groups.

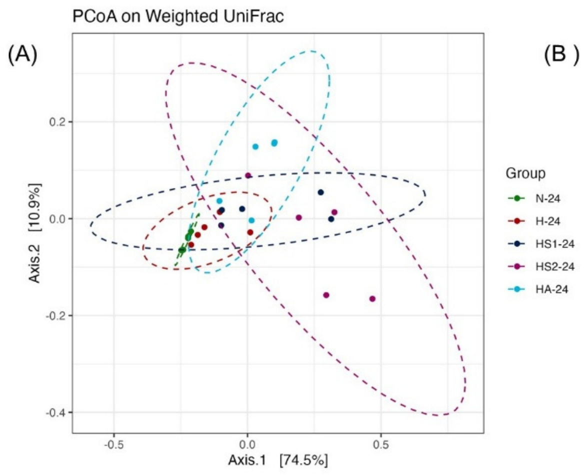

(B)

No.	pairs	P-value	P adjusted	sig
1	N-24 vs H-24	0.0110	0.0275	*
2	N-24 vs HS1-24	0.0050	0.0233	*
3	N-24 vs HS2-24	0.0030	0.0233	*
4	N-24 vs HA-24	0.0070	0.0233	*
5	H-24 vs HS1-24	0.0759	0.0949	
6	H-24 vs HS2-24	0.0180	0.0333	*
7	H-24 vs HA-24	0.0200	0.0333	*
8	HS1-24 vs HS2-24	0.2068	0.2298	
9	HS1-24 vs HA-24	0.4326	0.4326	
10	HS2-24 vs HA-24	0.0310	0.0442	*

Figure S5. Beta-diversity of fecal bacterial communities at week 24 based on weighted UniFrac distances. (A) Principal coordinates analysis (PCoA) plot and (B) PERMANOVA results. Rats fed a normal diet served as the negative control (N group), whereas HFD-fed rats served as the positive control (H group). Measurements were obtained after 8 weeks of intervention in obese rats administered with SCIINOs at 0.5 g/day (HS1 group), at 1 g/day (HS2 group), or in those treated with atorvastatin at 30 mg/kg/day (HA group). Accordingly, * indicates significant differences at $p < 0.05$.

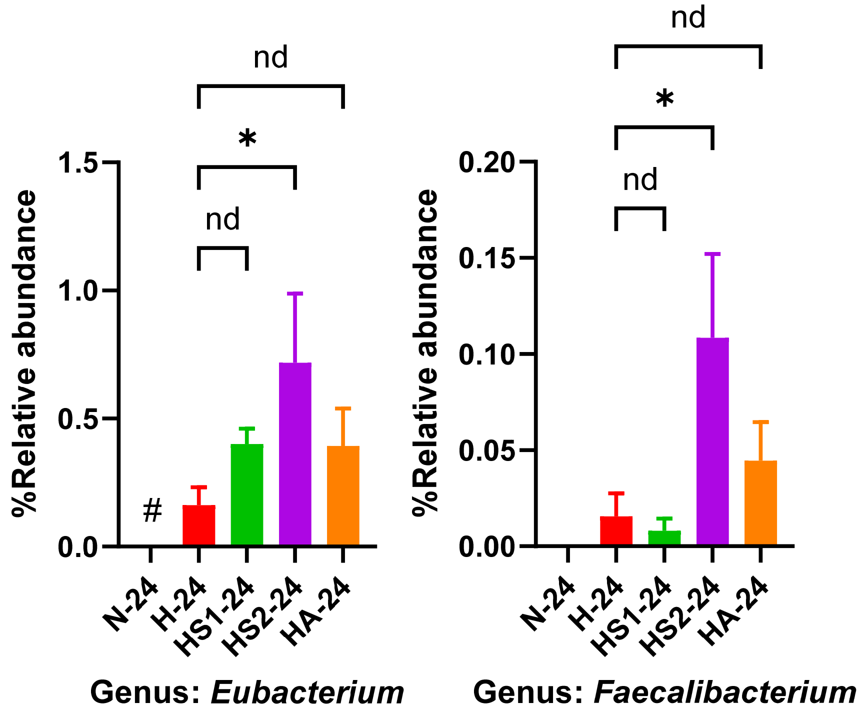

Figure S6. Multiple pairwise comparisons of *Eubacterium* and *Faecalibacterium* abundances across all groups. Pairwise comparisons between the N and H groups were performed using the Mann–Whitney U test, whereas multiple comparisons among the H, HS1, HS2, and HA groups were performed using the Kruskal–Wallis test (with pairwise post hoc comparisons as applicable). Data are presented as mean ± standard error of mean (SEM) (n = 5). * indicates $p < 0.05$; nd indicates no significant difference among H, HS1, HS2, and HA groups; # indicates significant difference between the N and H groups at $p < 0.05$, $p < 0.01$,.

Table S1. Composition of normal diet (ND) and high-fat diet (HFD).

Composition	Normal diet			Compositions	High fat diet		
	g	kcal	%E		g	kcal	%E
Carbohydrate	495.3	1981.2	51.99	Carbohydrate	190.76	763.04	14.27
Fat	83.7	753.3	19.77	Fat	342.24	3080.16	57.6
Protein	269	1076	28.24	Protein	353.6	1414.4	26.45
Vitamins	65.4		-	Cholesterol	10	90	1.68
Fiber	34.3	-	-	Vitamins	85.19	-	-
Total	947.7	3810.5	100	DL-Methionine	3	-	-
				Fiber	13.21	-	-
				Yeast powder	1	-	-
				Sodium chloride	1	-	-
				Total	1000	5347.6	100
